# *Wolbachia* and the insect immune system: what reactive oxygen species can tell us about the mechanisms of *Wolbachia*–host interactions

**DOI:** 10.3389/fmicb.2015.01201

**Published:** 2015-10-27

**Authors:** Roman Zug, Peter Hammerstein

**Affiliations:** Institute for Theoretical Biology, Humboldt-Universität zu Berlin, Berlin, Germany

**Keywords:** *Wolbachia*, reactive oxygen species, immune system, insects, symbiont–host interactions

## Abstract

*Wolbachia* are intracellular bacteria that infect a vast range of arthropod species, making them one of the most prevalent endosymbionts in the world. *Wolbachia*’s stunning evolutionary success is mostly due to their reproductive parasitism but also to mutualistic effects such as increased host fecundity or protection against pathogens. However, the mechanisms underlying *Wolbachia* phenotypes, both parasitic and mutualistic, are only poorly understood. Moreover, it is unclear how the insect immune system is involved in these phenotypes and why it is not more successful in eliminating the bacteria. Here we argue that reactive oxygen species (ROS) are likely to be key in elucidating these issues. ROS are essential players in the insect immune system, and *Wolbachia* infection can affect ROS levels in the host. Based on recent findings, we elaborate a hypothesis that considers the different effects of *Wolbachia* on the oxidative environment in novel vs. native hosts. We propose that newly introduced *Wolbachia* trigger an immune response and cause oxidative stress, whereas in coevolved symbioses, infection is not associated with oxidative stress, but rather with restored redox homeostasis. Redox homeostasis can be restored in different ways, depending on whether *Wolbachia* or the host is in charge. This hypothesis offers a mechanistic explanation for several of the observed *Wolbachia* phenotypes.

## Introduction

Reactive oxygen species (ROS) have long been viewed as purely harmful molecules contributing to oxidative stress, which can cause severe cell damage. On the other hand, ROS can also play a beneficial role, for example in intracellular signaling and innate immune defense. Intracellular bacteria of the genus *Wolbachia* have evolved intriguing capabilities to manipulate the biology of their arthropod hosts. *Wolbachia* have recently been shown to influence ROS production and the oxidative environment as a whole, suggesting an involvement of ROS in *Wolbachia*-induced phenotypes. In this article, we briefly review the relevant facts about ROS and give an overview of the insect immune response with a focus on ROS. In the main part, we outline the interactions between *Wolbachia* and the host immune system. We explore the possible roles of ROS in different *Wolbachia* phenotypes and hypothesize how interference with the host oxidative environment has shaped various aspects of the *Wolbachia-*insect symbiosis. Finally, we discuss some corollaries of the hypothesis.

## Reactive Oxygen Species, Oxidative Stress, and Redox Homeostasis

After the advent of molecular oxygen in the Earth’s atmosphere, organisms evolved metabolic mechanisms that use oxygen to produce energy. In eukaryotic cells, aerobic respiration takes place in the mitochondria. During oxidative phosphorylation, electrons are transferred along the mitochondrial respiratory chain to generate a proton gradient which eventually enables the synthesis of ATP. In this electron transport chain, the final acceptor of electrons is molecular oxygen which thereby is reduced to produce water. Occasionally, however, oxygen is prematurely and incompletely reduced, giving rise to superoxide. The superoxide anion belongs to a class of oxygen-derived molecules that readily oxidize other molecules and are commonly referred to as ROS. In addition to superoxide, ROS include hydrogen peroxide, hypochlorous acid, hydroxyl radical, singlet oxygen, and ozon. It has been known for a long time that an excessive load of ROS damages diverse cellular macromolecules, including proteins, lipids, and DNA, a process known as oxidative stress. The concept of oxidative stress has its roots in the mid-twentieth century when researchers began to explore the harmful effects of oxidizing free radicals (Gerschman et al., 1954) and their possible involvement in the aging process (Harman, 1956). A couple of years later, the antioxidant enzyme superoxide dismutase (SOD) was discovered which eliminates superoxide from the cell and thus protects the cell from its toxicity (McCord and Fridovich, 1969; McCord et al., 1971). Antioxidant enzymes aimed at detoxifying hydrogen peroxide include glutathione peroxidase (GPx), thioredoxin peroxidase (TPx), which is a member of the Peroxiredoxin (Prx) family, and catalase. Since the discovery of antioxidants, oxidative stress has been invoked as contributing to a number of diseases, including cancer (Storz, 2005; Valko et al., 2006), cardiovascular diseases (Griendling and FitzGerald, 2003), neurodegenerative disorders (Barnham et al., 2004), and diabetes (Maritim et al., 2003). Oxidative stress also negatively affects male and female reproductive ability, and detoxification of ROS by antioxidants is required for maintaining fertility/fecundity in *Drosophila* (Parkes et al., 1998), mosquitoes (DeJong et al., 2007), and mammals (Ho et al., 1998). In general, therefore, ROS have long been seen as harmful but unavoidable by-products of an aerobic lifestyle.

It therefore came as a surprise when enzymes were discovered whose sole function is the production of ROS (Royer-Pokora et al., 1986; Suh et al., 1999). The membrane-bound enzymes NADPH oxidases (NOX1-5) and dual oxidases (DUOX1–2) catalyze the reduction of molecular oxygen to generate superoxide and/or hydrogen peroxide, using NADPH as an electron donor (Brown and Griendling, 2009). DUOX enzymes can be distinguished from NOX enzymes by the presence of an extracellular peroxidase homology domain (PHD), in addition to the intracellular NADPH oxidase domain (Lambeth, 2004). Commonly, however, the term NOX is used for the whole seven-member protein family. NOX enzymes are expressed in a diverse array of cells and tissues and are present in most eukaryotes (Bedard et al., 2007; Aguirre and Lambeth, 2010). Therefore, the view that ROS are purely harmful by-products of mitochondrial metabolism needed reconsideration. It is important to note, though, that despite the existence of ROS-producing enzymes, the vast majority of cellular ROS (estimated at approximately 90%) can be traced back to a mitochondrial origin (Balaban et al., 2005). Nevertheless, the fact that ROS are actively synthesized prompted research into their possible biological functions. It is now clear that ROS, both those produced within mitochondria and those generated by NOX enzymes, act as important signaling molecules in diverse physiological processes. As such, ROS are involved in regulating cellular homeostasis, stem cell proliferation and differentiation, cell motility and migration, autophagy, cell death and aging, and, last but not least, immunity and host defense (D’Autréaux and Toledano, 2007; Hamanaka and Chandel, 2010; Finkel, 2011; Ray et al., 2012; Sena and Chandel, 2012; Nathan and Cunningham-Bussel, 2013; Holmström and Finkel, 2014; Lambeth and Neish, 2014; Schieber and Chandel, 2014; Reczek and Chandel, 2015). Therefore, organisms must tightly control the balance between ROS production and degradation. This fine-tuned balance between oxidants and antioxidants is called redox homeostasis.

## Insect Immunity: Antimicrobial Peptides, ROS, and Autophagy

The innate immune response of insects consists of multiple defense mechanisms, including epithelial barriers and both local and systemic immune reactions. Most research in insect immunity has focused on *Drosophila melanogaster* (Lemaitre and Hoffmann, 2007; Buchon et al., 2014; but see Rolff and Reynolds, 2009, for a broader perspective). The cellular immune response is executed by hemocytes and emcompasses several distinct mechanisms, including phagocytosis, encapsulation, coagulation, and melanization (Jiravanichpaisal et al., 2006; Lemaitre and Hoffmann, 2007; Strand, 2008; Fauvarque and Williams, 2011). Some of these mechanisms (encapsulation, melanization) involve the generation of ROS at infection sites to kill pathogens (Nappi et al., 1995; Nappi and Vass, 1998; Kumar et al., 2003). At the core of the systemic immune response lies the production of antimicrobial peptides (AMPs) by the fat body and their subsequent release into the hemolymph (for an overview of insect AMPs, see Yi et al., 2014). AMP gene expression is mainly controlled by two distinct signaling pathways, the Toll pathway and the Imd pathway, both of which include homologs of the NF-κB pathway (Khush et al., 2001; Brennan and Anderson, 2004; Ferrandon et al., 2007; Lemaitre and Hoffmann, 2007; Hetru and Hoffmann, 2009). The Imd pathway is predominantly activated by Gram-negative bacteria, whereas Gram-positive bacteria, fungi, and yeast trigger the Toll pathway (Buchon et al., 2014). In the lab, systemic responses have frequently been elicited by bacterial injection into the hemocoel. However, this might not reflect the natural way of infection. Commonly, epithelia such as those lining the gut are the first barrier a pathogen encounters when infecting the host. A peculiarity of gut epithelia is the fact that they not only are in constant contact with pathogens, but also host a number of beneficial commensal bacteria, the so-called gut microbiota. Commensal gut microbes are involved in diverse physiological functions of their hosts, including organ development and morphogenesis, host metabolism, and immunity (Sommer and Bäckhed, 2013; for reviews on the *Drosophila*/insect gut microbiota, see, for example, Buchon et al., 2013; Engel and Moran, 2013; Erkosar et al., 2013; Lee and Brey, 2013). The challenge for the host immune system, therefore, is to find the balance between fighting pathogens and tolerating the microbiota (Sansonetti and Medzhitov, 2009).

Accordingly, a tight regulation of the production of immune effector molecules is strictly needed. In the *Drosophila* gut, there are two major classes of immune effectors, AMPs and ROS (Ryu et al., 2010; Kuraishi et al., 2013). AMP generation in the gut is controlled by the Imd pathway, but not by the Toll pathway (Tzou et al., 2000). The Imd pathway is triggered when the bacterial cell wall component diaminopimelic acid (DAP)-type peptidoglycan (PG) is recognized by PG recognition proteins (PGRPs) in the host membrane (Leulier et al., 2003; Bosco-Drayon et al., 2012; Neyen et al., 2012; for reviews on PGRPs, see Royet and Dziarski, 2007; Royet et al., 2011). In the absence of pathogenic bacteria, PG-triggered AMP gene expression is repressed by negative regulators of the Imd pathway to protect the commensal microbiota, thereby maintaining the balance between immune tolerance and immune response (Lhocine et al., 2008; Ryu et al., 2008; Paredes et al., 2011; Bosco-Drayon et al., 2012; Bonnay et al., 2013; Dantoft et al., 2013).

Local production of AMPs only seems to constitute a complementary response against microbes that are resistant against ROS (Ryu et al., 2006), the second major immune effector class in the *Drosophila* gut. Indeed, DUOX-dependent production of microbicidal ROS serves as the first line of defense in gut immunity (Ha et al., 2005a, 2009a). It is assumed that the NADPH oxidase domain of DUOX synthesizes H_2_O_2_, which the PHD then converts into the highly microbicidal HOCl in the presence of chloride (Ha et al., 2005a). Infection-induced ROS generation in the *Drosophila* gut can also act as a signal for AMP production in the fat body, thus triggering a systemic immune response (Wu et al., 2012). After the pathogen-induced increase in ROS production, ROS levels are actively reduced by immune-regulated catalase (IRC) activity to avoid excessive oxidative stress (Ha et al., 2005b).

DUOX-dependent ROS production in the *Drosophila* gut is regulated by two signaling pathways (Bae et al., 2010): The enzymatic activity of DUOX is controlled by the Gαq-PLCβ-Ca^2+^ pathway (“DUOX activity pathway”; Ha et al., 2009a), while DUOX gene expression is regulated by a MEKK1-MKK3-p38-ATF2 pathway (“DUOX expression pathway”; Ha et al., 2009b, Chakrabarti et al., 2014). Activation of both pathways is required for stable ROS production. Interestingly, PG is able to activate the DUOX expression pathway, but not the DUOX activity pathway. Therefore, DUOX-dependent ROS generation cannot depend on PG alone (Ha et al., 2009a,b; Bae et al., 2010). Recently, bacterial-derived uracil was identified as a non-PG ligand triggering DUOX-dependent ROS generation (Lee et al., 2013). Uracil is probably recognized by a G-protein-coupled receptor (GPCR) and, via Hedgehog-induced signaling endosomes, induces PLCβ-dependent Ca^2+^ mobilization which triggers DUOX activation (Lee et al., 2015). Strikingly, uracil is released by pathogenic bacteria, but not by commensal symbionts (Lee et al., 2013). This allows the gut epithelia to distinguish between pathogens and commensal bacteria, thus maintaining immune homeostasis in the *Drosophila* gut (Kim and Lee, 2014; You et al., 2014).

What do we know about the involvement of ROS in innate immunity and host defense in insects other than *Drosophila*? The production of ROS as a countermeasure to bacterial and/or fungal infection has been reported from species as diverse as the cockroach *Blaberus discoidalis* (Blattodea; Whitten and Ratcliffe, 1999), the silkworm *Bombyx mori* (Lepidoptera; Ishii et al., 2008), the scale insect *Dactylopius coccus* (Hemiptera; García-Gil de Muñoz et al., 2007), the greater wax moth *Galleria mellonella* (Lepidoptera; Bergin et al., 2005), the sand fly *Lutzomyia longipalpis* (Diptera; Diaz-Albiter et al., 2012), the tiger moth *Parasemia plantaginis* (Lepidoptera; Mikonranta et al., 2014), and the cattle tick *Rhipicephalus microplus* (Ixodida; Pereira et al., 2001). Hematophagous insects may also become infected by blood–borne parasites, e.g., the malaria parasite *Plasmodium*. The mosquito *Anopheles gambiae* is one of the most efficient malaria vectors known. Interestingly, sufficiently high ROS levels are required for *An. gambiae* to mount an effective immune response against *Plasmodium* and bacteria (Kumar et al., 2003; Molina-Cruz et al., 2008). Elevated ROS levels to fight off *Plasmodium* can be generated by mitochondria in mosquito midgut cells (Gonçalves et al., 2012) or by an *Enterobacter* bacterium from the *An. gambiae* gut microbiota (Cirimotich et al., 2011). Therefore, host defense against bacterial, fungal, and *Plasmodium* infection based on ROS is widespread among various insect species.

Insect immunity based on the production of AMPs and ROS (controlled by the Toll pathway, the Imd pathway, and both DUOX pathways) is able to fight off both Gram-positive and Gram-negative bacteria, fungi, yeast, and protozoa such as *Plasmodium* (Carter and Hurd, 2010; Buchon et al., 2014). AMP production based on the Toll/Imd pathways may also be involved in the antiviral response, in addition to RNA interference and other mechanisms (Xi et al., 2008b; Sabin et al., 2010; Merkling and van Rij, 2013; Ferreira et al., 2014; Lamiable and Imler, 2014). To the best of our knowledge, however, nothing is known about ROS as antiviral effectors in a natural insect system (but see Wang et al., 2001, and below). In general, insect host defenses against intracellular pathogens (such as viruses) are less well studied than those against extracellular pathogens. AMPs have been shown to control obligate intracellular bacteria such as *Rickettsia* and *Anaplasma* (Baldridge et al., 2005; Liu et al., 2012b). Moreover, several immune responses are known that specifically target intracellular pathogens (Steinert and Levashina, 2011; Lundgren and Jurat-Fuentes, 2012; Péan and Dionne, 2014). Autophagy seems to represent a general and evolutionarily conserved defense mechanism against intracellular pathogens (Virgin and Levine, 2009; Deretic, 2010; Nakamoto et al., 2012; Yuk et al., 2012; Choy and Roy, 2013; Deretic et al., 2013). In *Drosophila*, for example, one type of PGRP (PGRP-LE) acts as an intracellular receptor for DAP-type PG and thus as an intracellular sensor of Gram-negative bacteria (Kaneko et al., 2006). PGRP-LE also induces an autophagic response to prevent the intracellular growth of bacterial pathogens, and this induction occurs independently of the Toll and Imd pathways (Yano et al., 2008; Kurata, 2010). Moreover, autophagy is also activated and regulated by ROS (Huang et al., 2009; Scherz-Shouval and Elazar, 2011; Sena and Chandel, 2012). In sum, several distinct and yet interconnected immune responses are at work to defend the insect host against a plethora of different pathogens.

## *Wolbachia* and the Insect Immune System

*Wolbachia* are maternally transmitted intracellular Gram-negative bacteria that infect a vast range of arthropod species, probably making them the most prevalent endosymbionts in the world (Hilgenboecker et al., 2008; Zug and Hammerstein, 2012; Weinert et al., 2015). *Wolbachia*’s evolutionary success is generally attributed to their reproductive parasitism, which ensures their vertical transmission from mother to offspring (Stouthamer et al., 1999; Werren et al., 2008). These reproductive manipulations include male killing, feminization, parthenogenesis induction, and cytoplasmic incompatibility (CI). The latter phenotype seems to be the most frequent one and occurs if males infected with CI-*Wolbachia* mate with uninfected females; these matings suffer from high offspring mortality. Infected females, in contrast, can mate successfully with both uninfected and infected males. In sum, all reproductive manipulations enhance the proportion of infected females and thus benefit the maternally inherited *Wolbachia*. Recently, however, two additional facets of the biology of *Wolbachia* are increasingly acknowledged to contribute to their success: horizontal transmission and mutualistic effects. Transmission between different host species is likely to be a major reason for *Wolbachia*’s vast abundance, particularly if it occurs over large phylogenetic distances (Zug et al., 2012). In addition, the global spread of *Wolbachia* might be facilitated by mutualistic effects such as increased host fecundity and longevity or protection against pathogens (Zug and Hammerstein, 2015). Nevertheless, reproductive parasitism seems to be more prevalent than mutualism in arthropod hosts, and it is frequently associated with a fitness cost to the host (Zug and Hammerstein, 2015).

What does the host defense against *Wolbachia* infection look like? In principle, hosts can employ two different strategies to defend themselves against infections: resistance and tolerance. Resistance is the ability to clear the infection, while tolerance is the ability to reduce the fitness costs of infection, without clearing the infection itself (Schneider and Ayres, 2008). Whether a host responds to *Wolbachia* through resistance or tolerance strongly depends on two features of the infection: its age and its phenotypic effects. A recently acquired infection is likely to trigger an immune response, which is the key resistance mechanism. In coevolved associations, by contrast, resistance may no longer be the best response to infection. Whether or not resistance is the host’s best option in coevolved symbioses mainly depends on the symbiont’s phenotype. Reproductive manipulations such as feminization and male killing reduce host fitness and thus are expected to lead to the evolution of resistance. Indeed, host suppressor alleles have been identified that confer resistance to feminizing and male killing *Wolbachia* (Rigaud and Juchault, 1992; Hornett et al., 2006). With other *Wolbachia* phenotypes, things are a bit more complex. In the case of CI, infected females are “addicted” to *Wolbachia*—if they lose the symbionts, their offspring will suffer from high mortality rates when fathered by infected males. Therefore, females infected with CI-*Wolbachia* are selected to maintain the bacteria and even increase the efficiency of maternal transmission. On the other hand, suppressor genes are predicted to spread in males, and successive selection for male suppressors of *Wolbachia* should lead to long-term elimination of infection (Koehncke et al., 2009). With respect to *Wolbachia*-induced parthenogenesis, the symbiont has gone to fixation in most populations that are infected. In these populations there are no males, and females depend on the bacteria for asexual reproduction. Under such circumstances of host dependence, infected females are not expected to evolve mechanisms of resistance (“dependence” barrier to resistance; Zug and Hammerstein, 2015). However, nuclear suppressor alleles have been hypothesized for populations where infected and uninfected individuals coexist (Huigens, 2003). Finally, if *Wolbachia* exhibits a mutualistic phenotype, evolution of resistance will also be selected against (“fitness benefit” barrier to resistance; Zug and Hammerstein, 2015). When resistance is not feasible, tolerance mechanisms represent an alternative host strategy to deal with the infection. The evolution of tolerance is associated with the attenuation of the immune response that originally was there to eliminate the bacteria. Immune tolerance is also an efficient means to reduce the risk that host tissue is damaged as a side effect of the immune response (immunopathology).

In summary, the evolution of host resistance is expected in many, but not all, *Wolbachia*–host associations. In those associations in which resistance evolution is expected, *Wolbachia* should, in principle, trigger the host immune system which should aim at eliminating the bacteria, regardless of whether they are novel or native. On the other hand, given the huge number of infected insect species and the recurrent occurrence of successful transmission into novel host species, why is the host defense machinery not more efficient in overcoming the infection? Have *Wolbachia* evolved mechanisms to suppress or interfere with the immune system, or do they hide from it? Or does the high prevalence of *Wolbachia* indicate that, frequently, hosts are not selected to evolve resistance (but rather tolerance)? In the following paragraphs, we outline in more detail the interplay between *Wolbachia* infection and the different host defense mechanisms, with special emphasis on the host oxidative environment.

### *Wolbachia* and AMP-/Autophagy-Based Immunity

**FIGURE 1:**
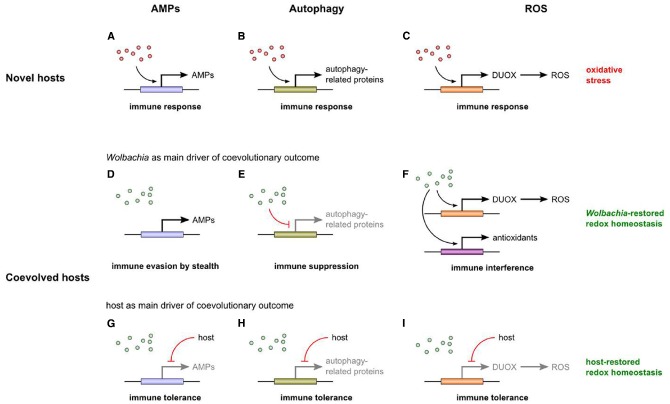
**Hypothesized effects of ***Wolbachia*** on the immune system of novel (A–C) and coevolved hosts (D–I).** Newly introduced *Wolbachia* (red dots) trigger an immune response by upregulating the expression of several immune effectors such as AMPs **(A)**, autophagy-related proteins **(B)**, and ROS **(C)**. A ROS-based immune response leads to oxidative stress. Due to host-symbiont coevolution, native *Wolbachia* (green dots) have ceased triggering an immune response. They neither induce nor suppress AMP expression, but evade the AMP-based immune response by stealth **(D)**. Presumably, they downregulate autophagy-related genes **(E)**. With regard to the ROS-based branch of the immune system, we hypothesize that *Wolbachia* not only induce ROS production and oxidative stress, but also the expression of antioxidant genes. By such immune interference, *Wolbachia* restore redox homeostasis **(F)**. Another coevolutionary outcome is host-driven shutdown of the immune response (immune tolerance; **G–I**). By evolving ROS-associated immune tolerance, the host restores redox homeostasis itself **(I)**. Note that evolution of resistance is also a possible outcome of coevolution, but eventually leads to symbiosis breakdown and therefore is not depicted here.

Interestingly, in their native hosts, *Wolbachia* do not induce AMP gene expression, as has been shown for *Aedes albopictus*, *D. melanogaster*, *Drosophila simulans*, and *Tetranychus urticae* (Bourtzis et al., 2000; Wong et al., 2011; Rancès et al., 2012; Zhang et al., 2015). On the other hand, *Wolbachia*-infected *D. simulans* and *Ae. albopictus* are still able to activate AMP gene expression when challenged by other bacterial pathogens, e.g., *E. coli* (Bourtzis et al., 2000). These results suggest that *Wolbachia* neither induce nor suppress the AMP-based branch of the immune system of their natural hosts. *Drosophila* species seem to be naturally infected with only two maternally inherited bacteria, *Wolbachia* and *Spiroplasma* (Mateos et al., 2006). In *Spiroplasma*-infected *D. melanogaster*, the same picture emerges: in their natural host, the bacteria neither upregulate nor downregulate the expression of AMP genes (Hurst et al., 2003; Hutchence et al., 2011). Taken together, these findings suggest that endosymbionts such as *Wolbachia* have evolved means to evade the host immune system by stealth (Figure 1D; Siozios et al., 2008). This notion is corroborated by the fact that, in the host cytoplasm, *Wolbachia* are located within vesicles whose outermost membrane is of host origin (Louis and Nigro, 1989). This probably helps the bacteria to hide from the host immune system. Another possible reason for the lack of *Wolbachia*-induced AMP upregulation is that the host has shut down the AMP-based immune response when selection favors the maintenance of the bacteria (Figure 1G). However, it is unclear how this immune tolerance could be restricted to *Wolbachia* so that other pathogens are still effectively targeted. This problem could be resolved by the fact that AMPs do not need to be shut down for ensuring immune tolerance in coevolved symbioses, but instead are actively involved in symbiont maintenance (Login et al., 2011).

The fact that *Wolbachia* do not elicit an AMP-based immune response in their native hosts stands in stark contrast to the strong induction of AMP gene expression when *Wolbachia* are introduced into novel hosts (Figure 1A; Xi et al., 2008a; Kambris et al., 2009, 2010; Moreira et al., 2009; Bian et al., 2010). This is indicative of a systemic immune response triggered by the canonical Toll and/or Imd pathway (immune upregulation; note that the term immune priming is equivalent to such general immune upregulation only in its unspecific meaning; compare, for example, Roth et al., 2009 and Masri and Cremer, 2014 for a different usage of the term). As Gram-negative bacteria, newly introduced *Wolbachia* are probably detected by the Imd pathway that is triggered by recognition of DAP-type PG from the bacterial cell wall. Although *Wolbachia* lack a proper cell wall and PG has never been detected, they are probably able to synthesize DAP (Dunning Hotopp et al., 2006; Vollmer et al., 2013). Moreover, it was recently shown that the PG-associated lipoprotein (PAL) is located on the cell membrane of *Wolbachia* (Voronin et al., 2014). PAL is known to specifically bind DAP (Parsons et al., 2006). Therefore, DAP is present on the *Wolbachia* membrane, and perhaps this is sufficient to be recognized by PGRPs which then trigger the Imd pathway and subsequent AMP generation.

The discovery of PGRP-LE as an intracellular sensor of DAP-type PG (Kaneko et al., 2006) also opens the possibility of an autophagic immune defense against *Wolbachia*. It was recently shown that *Wolbachia* induce the autophagy pathway in a naturally infected *Ae. albopictus* cell line (Voronin et al., 2012). Hence, one might expect bacterial strategies to counteract autophagy. Indeed, autophagy-associated genes are downregulated in the ovaries of two hosts naturally infected with *Wolbachia*, the woodlouse *Armadillidium vulgare* and the wasp *Asobara tabida*, supporting the notion that the symbionts suppress the autophagic signal to prevent their elimination (Figure 1E; Chevalier et al., 2012; Kremer et al., 2012). Again, it is also conceivable that the host itself is responsible for the downregulation—another possible case of evolved immune tolerance when symbiont presence is favored (Figure 1H). In contrast to these coevolved associations, a transfected *Wolbachia* strain causes a catastrophic autophagic response in another woodlouse, *Porcellio d. dilatatus*, resulting in the death of the new host (Figure 1B; Le Clec’h et al., 2012). Therefore, an autophagic immune response is observable in novel, but not in native hosts, mirroring the situation with regard to AMP-based immune defense.

How can we reconcile these differing findings concerning the immune response to *Wolbachia* in native vs. novel hosts? Perhaps, it is not too surprising that *Wolbachia* do elicit an immune response in novel hosts. In insects that acquired *Wolbachia* only recently (either by natural or artificial means), the bacteria are recognized as foreign, probably by PGRPs detecting DAP on *Wolbachia* membranes, and then AMP- and/or autophagy-associated defense mechanisms are triggered to eliminate the infection (Figures 1A,B). With ongoing coevolution, however, *Wolbachia* have found ways to prevent their elimination, for example by evading the AMP-based immune response (immune evasion by stealth; Figure 1D) and by suppressing the autophagy-associated immune defense (immune suppression; Figure 1E). Alternatively, evolution of immune tolerance enables the host to reduce costly defense mechanisms when selection favors the presence of *Wolbachia* (Figures 1G,H).

### *Wolbachia* and ROS-Based Immunity

Given their vertical transmission through the female germline and their reproductive manipulations, *Wolbachia* are expected to reside primarily in the host reproductive tissues. Although this is true, they are also able to infect somatic tissues, including tissues of immunological importance, such as the gut, fat body, and hemolymph (Dobson et al., 1999; Cheng et al., 2000; Ijichi et al., 2002; Zouache et al., 2009; Frost et al., 2014). In gut epithelia, AMPs represent only one of two major classes of immune effectors, the other one being ROS (see above). Therefore, when asking about the relationship between *Wolbachia* infection and the host immune system, it is also important to consider possible interactions between *Wolbachia* on the one hand and ROS and the oxidative environment on the other hand, in particular if infection in the gut has been reported.

The first, indirect evidence of such an interaction between *Wolbachia* and the host oxidative environment came from studies on the role of mitochondria in various aspects of host biology. In *D. melanogaster*, tetracycline treatment to eliminate *Wolbachia* resulted in a significant decrease in lipid hydroperoxide, a marker for ROS-induced oxidative damage (Driver et al., 2004). However, this finding could be due to a direct negative effect of tetracycline on mitochondrial efficiency (Ballard and Melvin, 2007). Further indirect evidence comes from the fact that *Wolbachia* infection can have a profound influence on mitochondrial DNA (mtDNA) haplotype diversity (Hurst and Jiggins, 2005), and different mtDNA haplotypes can differ in mitochondrial ROS production rates (Ballard, 2005).

Brennan et al. (2008) were the first to demonstrate a more direct effect of *Wolbachia* on the host oxidative environment. The mosquito *Ae. albopictus* is naturally infected with CI-inducing *Wolbachia*. In an *Ae. albopictus* cell line, the authors found that *Wolbachia* infection is associated with high levels of ROS (as compared to an identical cell line treated with the antibiotic rifampicin). These ROS probably are a product of the host immune response (although they may also be a side-product of bacterial metabolism). In addition, *Wolbachia* infection is associated with the upregulation of several host antioxidant genes. These antioxidant proteins include copper-zinc SOD1, Prx5, and GPx. Antioxidant upregulation may be a host countermeasure to mitigate the negative effects of increased ROS levels. However, as the authors point out, there is so far only little support in the literature for antioxidant upregulation as a host response to pathogen infection. Alternatively, one might speculate that *Wolbachia* induce the host antioxidant system in order to be protected against the host immune response based on increased ROS levels. Possibly, induction of the host antioxidant system is due to effectors secreted by the bacterial type IV secretion system (T4SS). The *Wolbachia* T4SS is a potential pathway to transfer effector proteins into the host cytoplasm and therefore might be involved in *Wolbachia*-induced host phenotypes (Pichon et al., 2009). Recently, a T4SS effector in *Ehrlichia* (a close relative of *Wolbachia*) was shown to be translocated to mitochondria and to upregulate a host SOD (MnSOD), thereby reducing ROS levels and apoptosis (Liu et al., 2012a). Lastly, *Wolbachia* also seem to be able to produce their own antioxidants to protect themselves, since two bacterial antioxidant proteins were identified as well, a bacterial type of SOD (Fe-SOD) and bacterioferritin (Bfr). Iron (Fe) is an essential element for most organisms, but also a cause of oxidative stress as it catalyzes the generation of highly reactive hydroxyl radicals (Fenton reaction; Nappi and Vass, 2002). Hence, bacterioferritin has important functions both in bacterial iron storage and, although not commonly referred to as an antioxidant, in fighting iron-mediated oxidative stress (Carrondo, 2003). Upregulation of *Wolbachia* bacterioferritin expression under iron-induced stress was also observed in naturally infected *D. simulans* (Kremer et al., 2009). Given the crucial role of iron at the interface of immunity, infection and host–pathogen interactions (Cassat and Skaar, 2013; Nairz et al., 2014), *Wolbachia*’s ability to interfere with host iron metabolism might be an important factor underlying diverse phenotypes and thus contributing to the unparalleled success of *Wolbachia* (Gill et al., 2014).

#### *Wolbachia* and the Oxidative Environment: A Hypothesis

Based on the results by Brennan et al. (2008), on subsequent propositions regarding the possible involvement of *Wolbachia* in the host oxidative environment (Kremer et al., 2010; Moné et al., 2014), and on the findings concerning AMP-/autophagy-based immunity, we propose the following hypothesis. In novel hosts, *Wolbachia* induce a ROS-based immune response, leading to oxidative stress (Figure 1C). In general, therefore, we expect infections in novel hosts to be associated with a disruption of redox homeostasis (although *Wolbachia* effects on antioxidant production are hardly predictable). In native hosts, by contrast, *Wolbachia* infection is expected to be associated with restored redox homeostasis, resulting from coevolutionary processes between symbiont and host. Redox homeostasis can be restored by *Wolbachia* or by the host (or by a combination of both) because both benefit from reduced oxidative stress. In the first case, *Wolbachia* not only induce a ROS-based immune response, but also the expression of antioxidant genes (regardless of whether these genes are part of the symbiont or host genome). In doing so, the bacteria interfere with the host immune response (immune interference) and are involved in maintaining redox homeostasis (Figure 1F). This may be particularly relevant if there are additional sources of oxidative stress (e.g., iron overload). In the second case, the host decreases the *Wolbachia*-induced immune response by reducing ROS production or by increasing antioxidant production (immune tolerance), and thereby restores redox homeostasis itself (Figure 1I). In what follows, we will gather further evidence in support of this hypothesis.

#### *Wolbachia* and the Oxidative Environment in Novel Hosts

Several studies report effects of *Wolbachia* infection on the oxidative environment of arthropods that are naturally either uninfected of infected with a different strain. Examples where a novel *Wolbachia* infection causes an increase in ROS levels include the mosquitoes *Aedes aegypti* (Pan et al., 2012), *Aedes polynesiensis* (Andrews et al., 2012), and *Anopheles stephensi* (Bian et al., 2013). All mosquitoes were transfected with the *Wolbachia* strain *w*AlbB, which naturally infects *Ae. albopictus*. In *Ae. aegypti*, ROS production was shown to be due to the upregulation of NADPH oxidase (NOXM) and dual oxidase (DUOX2), the latter one being upregulated 28-fold (Pan et al., 2012). Interestingly, the authors found that increased ROS levels activate the Toll pathway, leading to the production of AMPs and antioxidants. The fact that *Wolbachia* induce both the activation of ROS and antioxidants in *Ae. aegypti* is reminiscent of the situation in evolved symbioses (immune interference by *Wolbachia*). Given the relatively close phylogenetic relationship between donor (*Ae. albopictus*) and recipient (*Ae. aegypti*), it might not be too difficult for *w*AlbB to induce antioxidant production in *Ae. aegypti* and thus establish redox homeostasis in a novel host. On the other hand, some studies involving transfected cell lines show the downregulation of antioxidants as a result of infection (Xi et al., 2008a; Hughes et al., 2011). In sum, there is good evidence of the induction of ROS production by *Wolbachia* in novel hosts, whereas findings on the effects of novel infections on antioxidant production are so far inconclusive.

#### *Wolbachia* and the Oxidative Environment in Native Hosts

In addition to the results by Brennan et al. (2008) in an *Ae. albopictus* cell line, there is also evidence in support of our hypothesis that comes from whole insects. Using different methods, Molloy and Sinkins (2015) re-examined the production of ROS and antioxidants in *Ae. albopictus*, both in mosquito and cell lines. Interestingly, they did not find any significant difference in infected vs. uninfected lines, a finding that differs from that by Brennan et al. (2008). Nevertheless, it can similarly be interpreted as an outcome of host-symbiont coevolution, i.e., as attenuation of the immune response to *Wolbachia* in its natural host (immune tolerance). Thus, although it is unclear why both studies come to different results at the molecular level, the conclusion that can be drawn from them is the same: coevolution between *Ae. albopictus* and its *Wolbachia* has led to restored redox homeostasis, either through immune interference (suggested by the results from Brennan et al., 2008) or immune tolerance (suggested by Molloy and Sinkins, 2015).

In *D. simulans* naturally infected with *Wolbachia*, total ROS levels are significantly higher in infected males than in males cured of infection. Moreover, DUOX is located in close proximity to the *Wolbachia*-containing vesicles (Haukedal, 2013). This suggests that the host recognizes *Wolbachia* as foreign and prompts an immune response involving DUOX-dependent ROS production. On the other hand, total SOD levels (including two host SODs and bacterial Fe-SOD) are also significantly higher in infected flies than in uninfected flies (Brennan et al., 2012). These findings suggest that *Wolbachia* infection in the natural host *D. simulans* induces not only a host immune response, but also antioxidant production.

As already mentioned, the ability of *Wolbachia* to interfere with the host oxidative environment might be of particular importance if the level of oxidative stress is elevated by external factors. Toxicity of the heavy metal lead is mainly attributed to its ability to generate ROS and to impair the antioxidant defense (Flora et al., 2012). When *D. melanogaster* is challenged by a lead-contaminated diet, flies cured of infection exhibit a strongly increased malondialdehyde content, which is a marker for oxidative stress. In addition, high-lead diet significantly decreases SOD activity in cured flies, but not in infected flies (Wang et al., 2012).

Another example of the putative role of *Wolbachia* in maintaining redox homeostasis under stressful conditions involves the oxidative challenge imposed by blood-feeding. Ingestion of a blood meal is associated with the release of large amounts of the iron-containing cofactor heme in the gut. When not bound to proteins, heme has potential pro-oxidant and cytotoxic effects in that it converts weakly ROS into highly reactive ones (Jeney et al., 2002). Hematophagous insects have evolved different mechanisms to be protected from these cytotoxic effects, including the binding, aggregation, and degradation of heme, and expression of antioxidant enzymes (Oliveira et al., 1999; Graça-Souza et al., 2006; Paiva-Silva et al., 2006). Maintenance of redox homeostasis in the midgut after a blood meal is crucial, not least because of the pivotal role of ROS in gut immunity. In the mosquito *Ae. aegypti*, a blood meal leads, perhaps counterintuitively at first, to a dramatic decrease in ROS levels in the midgut (Oliveira et al., 2011). This decrease is due to a heme-mediated activation of protein kinase C (PKC) which leads to lowered ROS generation in midgut epithelial cells. The authors interpret this as an adaptation to compensate for the pro-oxidant blood meal and to avoid heme-mediated oxidative stress, thus maintaining redox homeostasis. However, lowered ROS levels in the gut are probably associated with decreased resistance to infection and increased mortality (Oliveira et al., 2011). Interestingly, overall ROS levels do not change significantly after a blood meal in *Ae. polynesiensis* which, unlike *Ae. aegypti*, is naturally infected with *Wolbachia* (Andrews et al., 2012). In a coevolutionary process, the host might have abolished the PKC-mediated decrease in ROS levels in the gut because of *Wolbachia*-induced antioxidant production (immune interference). Therefore, it is reasonable to assume that *Wolbachia* help in maintaining redox homeostasis in this evolved symbiosis (Gill et al., 2014). Moreover, when *Ae. polynesiensis* is fed sucrose only, there is no significant difference between ROS levels of infected and cured mosquitoes, and these ROS levels are lower than that of artificially infected mosquitoes (Andrews et al., 2012). A possible explanation for this finding is that, due to coevolution between *Ae. polynesiensis* and its symbiont, the mosquito has reduced ROS production to mitigate oxidative stress (immune tolerance). Evolution of immune tolerance might therefore also be at play in the *Wolbachia*–*Ae. polynesiensis* symbiosis (Moné et al., 2014).

In the spider mite *T. urticae*, *Wolbachia* infection is associated with the enrichment of gene sets related to oxidoreductase activity (Zhang et al., 2015). Oxidoreductases are known to produce ROS (Raha and Robinson, 2000; Esterházy et al., 2008), but also to control redox homeostasis (Messens et al., 2013). Moreover, *Wolbachia* encodes an oxidoreductase (*α*-DsbA1) which, due to its low redox potential, might have antioxidant properties (Kurz et al., 2009). Therefore, it is conceivable that *Wolbachia* directly or indirectly regulate redox homeostasis and thus maintain their association with *T. urticae*.

We do not want to conceal that there also are some findings from natural *Wolbachia*–host associations that are more difficult to reconcile with the above hypothesis, or at least more difficult to interpret. In the pill bug *Armadillidium vulgare*, some antioxidants (thioredoxin, ferritin) are upregulated in the ovaries of infected individuals (as compared to uninfected ones), while others (Prx, glutathione peroxidase) are downregulated (Chevalier et al., 2012). Perhaps, upregulation of some antioxidants is just a compensation for the downregulation of others, or *vice versa*. It is unclear, however, what is induced by the host and what by the bacteria. In the parasitoid wasp *Asobara tabida*, the expression of several antioxidant genes (oxidoreductase, glutathione peroxidase, ferritin) is downregulated in infected ovaries, compared to ovaries from cured wasps (Kremer et al., 2012). At first, this seems to contradict our hypothesis. However, the *Wolbachia–Asobara tabida* association is a special case because here, the host is strictly dependent on its symbiont (Zug and Hammerstein, 2015). Females cured of infection fail to produce oocytes, due to extensive apoptosis in egg chambers (Pannebakker et al., 2007). The authors suggest a co-evolutionary scenario where the wasp responds to infection with apoptosis, which is then suppressed by *Wolbachia*. *Asobara tabida* in turn compensates for suppression by further increasing the apoptotic signal because it is essential for proper egg development (Pannebakker et al., 2007). There is good empirical support for this scenario. First, *Wolbachia* are probably able to directly or indirectly suppress apoptosis. Suppression of apoptosis might be due to *Wolbachia* interfering with host iron metabolism and oxidative stress control (Kremer et al., 2010; Gill et al., 2014; Zug and Hammerstein, 2015). Moreover, it is known that ROS can act as initiators and mediators of apoptosis (Simon et al., 2000; Dixon and Stockwell, 2014). Therefore, downregulation of antioxidant genes could be a host measure to further increase the apoptotic signal. In sum, the dependence of *Asobara tabida* on *Wolbachia* might well be a consequence of the evolution of tolerance following the disruption of redox homeostasis (Moné et al., 2014; Zug and Hammerstein, 2015).

Lastly, we point to the fact that all cases that are compatible with the hypothesis involve CI-inducing *Wolbachia* (*Ae. albopictus*, *Ae. polynesiensis*, *D. melanogaster*, *D. simulans*, *T. urticae*). In contrast, *Armadillidium vulgare* is naturally infected with feminizing *Wolbachia*, and the strain that *Asobara tabida* depends on for oogenesis does not exhibit any reproductive phenotype (although the possibility that it induces CI remains untested). Therefore, one could think of a mechanistic connection between the host oxidative environment and the CI phenotype. Indeed, Brennan et al. (2012) showed that total SOD levels are significantly higher in testes of *D. simulans* males infected with CI-*Wolbachia* than in testes of cured males. Taking this as evidence of higher oxidative stress in infected testes, the authors presumed that disruption of redox homeostasis caused DNA damage in spermatocytes of infected males. Strikingly, DNA damage is significantly higher in infected compared to uninfected spermatocytes and might be a contributing factor to the sperm modification characteristic of CI (Brennan et al., 2012). For example, DNA damage in spermatocytes could, after fertilization, lead to DNA replication defects in the male pronucleus as observed in CI crosses in *D. simulans* (Landmann et al., 2009).

### *Wolbachia* and Anti-Pathogenic Effects

The possibility of *Wolbachia*-induced host protection has recently spurred intense research efforts. Taking a slightly critical stance, we have proposed to distinguish protection from mere anti-pathogenic effects (Zug and Hammerstein, 2015). Following our definition, *Wolbachia* are said to induce an antipathogenic effect whenever infection increases host resistance and/or tolerance to pathogens. However, an antipathogenic effect should only be classified as protection if it is associated with a fitness benefit to the host. Since so far only a few studies have found evidence for *Wolbachia*-mediated protection in the field (Hedges et al., 2008; Teixeira et al., 2008; Osborne et al., 2009; Zélé et al., 2012), we here focus on antipathogenic effects.

The molecular mechanisms underlying *Wolbachia*-mediated antipathogenic effects are still unclear (Rainey et al., 2014; Johnson, 2015; Zug and Hammerstein, 2015). Antiviral effects seem to be more frequent than antibacterial effects. Moreover, the strength of the antipathogenic effect is positively correlated to *Wolbachia* density. But how is the insect immune system involved? Antipathogenic effects are frequently observed when *Wolbachia* are transfected into hosts that are either naturally uninfected or infected with a different strain. As outlined above, in such cases, infection induces the upregulation of host immune genes, in particular genes involved in the Toll and Imd pathway, leading to the generation of AMPs. Such immune upregulation of Toll/Imd pathway genes is assumed to underlie antipathogenic effects in novel hosts, especially antiviral effects in mosquitoes (Xi et al., 2008a, Kambris et al., 2009, 2010; Moreira et al., 2009; Bian et al., 2010; Pan et al., 2012). However, other studies have shown that, both in native and novel hosts, genes involved in the Toll or Imd pathway are not required for *Wolbachia*-mediated antipathogenic effects (Wong et al., 2011; Rancès et al., 2012, 2013; Chrostek et al., 2014; Ferreira et al., 2014; Martinez et al., 2014). Therefore, upregulation of immune genes involved in the Toll/Imd pathways cannot be the universal explanation for *Wolbachia*-induced antipathogenic effects, let alone for host protection in the field (Zug and Hammerstein, 2015).

The possible role of ROS in *Wolbachia*-induced antipathogenic effects has been less intensively studied than that of AMPs. The mosquito *Ae. aegypti* is naturally not infected with *Wolbachia*, but transfection of the *w*AlbB strain into *Ae. aegypti* inhibits replication of Dengue virus (Bian et al., 2010). It could be shown that transfection induces NOX- and DUOX-dependent ROS generation. Increased ROS levels activate the Toll pathway, which then mediates the production of antioxidants and AMPs such as defensin and cecropin. These AMPs are involved in inhibiting the proliferation of Dengue virus in *Wolbachia*-transfected mosquitoes (Pan et al., 2012). In transfected *Ae. albopictus* mosquitoes, by contrast, ROS-mediated immune activation is probably not involved in the antiviral effect of *Wolbachia* (Molloy and Sinkins, 2015). A recent study analyzed the relationship between ROS levels and antiviral effects in naturally infected *Drosophila* strains (Wong et al., 2015). The study included *Wolbachia* strains that were known to either have an antipathogenic effect (“protective” strains) or not (“non-protective” strains). In flies that harbor a protective strain, ROS levels are significantly higher than in flies cured of the protective strain. By contrast, presence of the non-protective strain has no significant effect on ROS levels relative to cured flies. These findings suggest that ROS levels are increased in *Drosophila* naturally infected with protective *Wolbachia* strains. Moreover, elevated ROS levels confer a survival advantage against mortality induced by Drosophila C virus (DCV; Wong et al., 2015). The anti-DCV effect is probably not mediated by the Toll pathway because *Wolbachia*-induced antiviral effects were shown to be independent of this pathway in *Drosophila* for both Dengue virus and DCV (Rancès et al., 2013; Ferreira et al., 2014). Interestingly, the ROS-mediated survival advantage is not associated with reduced virus accumulation, pointing to increased tolerance rather than resistance (Wong et al., 2015). Tolerance mechanisms have been shown to be at play in other coevolved *Wolbachia*–host systems where the symbionts induce antipathogenic effects (Teixeira et al., 2008; Osborne et al., 2009; Zélé et al., 2014). In sum, the possibility that a *Wolbachia*-induced ROS-based immune response is involved in antipathogenic effects constitutes a promising topic for future research.

### *Wolbachia*, ROS, Life-History Trade-Offs, and Mitohormesis

Organisms cannot maximize all fitness-relevant traits at once. Rather, they face the challenge to optimally allocate limited resources among those traits. Hence, the evolution of fitness-related traits is constrained by the existence of trade-offs between them. These trade-offs play a fundamental role in life-history theory (Stearns, 1989). Along these lines, immune defense can be viewed as a life-history trait as well, and trade-offs between immunity and other fitness-related traits (“costs of immunity”) have been gaining increasing attention among evolutionary ecologists (Sheldon and Verhulst, 1996; Zuk and Stoehr, 2002; Schmid-Hempel, 2003; Schulenburg et al., 2009; McKean and Lazzaro, 2011).

Much effort has been made to elucidate the physiological mechanisms underlying life-history trade-offs. Given their antagonistic and pleiotropic effects, ROS have recently been proposed as central players in the occurrence of such trade-offs (Dowling and Simmons, 2009; Monaghan et al., 2009; Metcalfe and Alonso-Alvarez, 2010; Isaksson et al., 2011; but see Speakman and Garratt, 2014). In particular, because of their pivotal role in innate immunity on the one hand and in oxidative stress on the other hand, ROS may be a key factor underlying the trade-off between immunity and other life-history traits such as fecundity and longevity (Moné et al., 2014).

Building upon these ideas and on the intimate connections between *Wolbachia* and the host oxidative environment, one may speculate that *Wolbachia* are involved in the occurrence of the trade-off between immunity and other life-history traits, and that this involvement is, at least in part, mediated by ROS. There is some evidence for this hypothesis. Pigeault et al. (2014) studied the effect of transfected *Wolbachia* strains on immunity and reproduction in the woodlouse *Porcellio dilatatus*. They found a clear trade-off between both life-history traits: the *w*Con strain increases investment in immune parameters but reduces reproductive investment (whereas the *w*Dil strain has the converse effect). However, the tested immune parameters (such as hemocyte density or phagocytosis activity) do not allow to draw a conclusion on whether ROS are involved in the trade-off. In *D. simulans*, there is a similar trade-off between *Wolbachia*-induced antiviral protection and egg hatch rates, female fecundity, and male fertility (Martinez et al., 2015). Another example of *Wolbachia*-associated costs of immunity involves the trade-off between immunity and longevity. *Wolbachia* strains that induce strong antiviral effects in *D. melanogaster* (so-called *w*MelCS-like strains) often shorten the host lifespan (Chrostek et al., 2013). Strikingly, the *w*MelCS strain was recently shown to increase ROS concentration twofold relative to a *Wolbachia*-free control (Wong et al., 2015). Therefore, it is possible that elevated ROS levels are responsible not only for the antiviral effect, but also for the shortened lifespan.

The impact of ROS and oxidative stress on longevity and aging has been debated for more than half a century. The seminal “free radical theory of aging” states that the production of mitochondrial ROS is the major cause of aging (Harman, 1956, 1972; Balaban et al., 2005). However, findings are accumulating that seem to be incompatible with this theory (Lapointe and Hekimi, 2010; Speakman and Selman, 2011; Stuart et al., 2014; but see Kirkwood and Kowald, 2012). In particular, recent evidence suggests that moderately increased formation of ROS in the mitochondria causes higher stress resistance and eventually extends life span, a process that has been termed mitochondrial hormesis (mitohormesis; Ristow and Schmeisser, 2014; Yun and Finkel, 2014). In general, hormesis is defined as any adaptive response exhibiting a biphasic dose response (Calabrese and Baldwin, 2002). Usually, such biphasic dose responses are characterized by a beneficial effect at low doses and a harmful effect at higher doses. In a narrower, and recently more frequently used, sense, hormesis describes the phenomenon that a mild, sublethal stress causes an adaptive response that protects against larger subsequent stresses. The latter meaning of the term has been named “stress-response hormesis” (Gems and Partridge, 2008). Mitohormesis represents a form of stress-response hormesis: Mild mitochondrial stress increases ROS formation which induces stress response mechanisms (such as antioxidant production), ultimately causing a long-term reduction of oxidative stress. Mitohormesis thus involves both an increase in mitochondrial ROS and a subsequent antioxidant response, and the notion of a mitohormetic pathway is tightly associated with the role of ROS as important signaling molecules (Hamanaka and Chandel, 2010; Finkel, 2011). Several recent studies have shown this mitohormetic pathway to be at work in promoting survival and longevity (Kharade et al., 2005; Chávez et al., 2007; Schulz et al., 2007; Zarse et al., 2012; Mouchiroud et al., 2013; De Haes et al., 2014). Given that *Wolbachia* are known to promote longevity in several hosts (Zug and Hammerstein, 2015), it is tempting to speculate that they do so by triggering the mitohormetic pathway. More generally, the mitohormetic pathway is strongly reminiscent of the hypothesized “immune interference” phenotype of *Wolbachia* in native hosts (in which the symbionts not only induce a ROS-based immune response, but also the expression of antioxidant genes; Figure 1F). Taken together, some fitness-enhancing effects of native *Wolbachia* (e.g., promoting longevity, maintaining redox homeostasis) might be attributable to mitohormesis.

With regard to the impact of ROS on fitness-related traits, the trade-off approach and the mitohormesis approach might appear to come to quite different conclusions. For example, ROS are assumed to shorten lifespan under the former approach and to extend lifespan under the latter. More generally, the trade-off approach states that *Wolbachia* (via ROS) have a positive effect on some fitness parameters and a negative effect on others, whereas the mitohormesis approach emphasizes the positive fitness effect of *Wolbachia*-induced mitochondrial ROS formation. However, hormesis itself is assumed to trade off with at least some fitness-related traits because a positive hormetic effect on overall fitness would be at odds with life-history theory (Forbes, 2000). Accordingly, a recent study finds that pathogen challenge in *Drosophila* enhances not only survival and fecundity, but also susceptibility to infection, suggesting a trade-off between hormesis and immunity (McClure et al., 2014). Therefore, both approaches involve some form of trade-off and thus are not mutually exclusive.

## Conclusion

Reactive oxygen species represent a double-edged sword: They are known to cause oxidative stress and damage cellular macromolecules. However, given their cytotoxic nature, ROS also are efficient microbicidal effectors which play a crucial role in the insect immune system. Due to this antagonistic pleiotropy, ROS probably underlie evolutionary trade-offs between immunity and other life-history traits such as fecundity and longevity. *Wolbachia* are widespread intracellular bacteria famous for their ability to modulate exactly these fitness-related host traits in intriguing ways. At the same time, they must be able to cope with the host immune system in order to invade and persist in their insect hosts. Therefore, the host oxidative environment represents a promising area to elucidate the mechanisms of *Wolbachia*–host interactions.

In newly infected hosts, *Wolbachia* usually trigger an immune response which is aimed at eliminating the infection. In co-evolved associations, by contrast, either the host has curbed the immune response when it pays to do so, or the symbionts have evolved ways to resist the host immune response. They do so by adopting a variety of strategies, including immune evasion by stealth, suppression, and interference. We propose that in co-evolved symbioses, *Wolbachia* frequently make use of the latter strategy in that they not only induce a ROS-based immune response but also an antioxidant response. Thereby the bacteria are involved in maintaining redox homeostasis. Interference with the host oxidative environment might also underlie other mutualistic phenotypes of *Wolbachia* such as enhancing host defense or promoting longevity, possibly via mitohormetic effects. On the other hand, *Wolbachia*-induced ROS formation might be involved in parasitic phenotypes such as cytoplasmic incompatibility. Taken together, *Wolbachia*’s impact on the host oxidative environment probably contributed to their tremendous success and opens up exciting avenues for future research.

### Conflict of Interest Statement

The authors declare that the research was conducted in the absence of any commercial or financial relationships that could be construed as a potential conflict of interest.

## References

[B1] Aguirre J. Lambeth J. D. (2010). Nox enzymes from fungus to fly to fish and what they tell us about Nox function in mammals. Free Radic. Biol. Med. 49, 1342–1353. 10.1016/j.freeradbiomed.2010.07.027 20696238 PMC2981133

[B2] Andrews E. S. Crain P. R. Fu Y. Howe D. K. Dobson S. L. (2012). Reactive oxygen species production and *Brugia pahangi* survivorship in *Aedes polynesiensis* with artificial *Wolbachia* infection types. PLoS Pathog. 8:e1003075. 10.1371/journal.ppat.1003075 23236284 PMC3516568

[B3] Bae Y. S. Choi M. K. Lee W.-J. (2010). Dual oxidase in mucosal immunity and host–microbe homeostasis. Trends Immunol. 31, 278–287. 10.1016/j.it.2010.05.003 20579935

[B4] Balaban R. S. Nemoto S. Finkel T. (2005). Mitochondria, oxidants, and aging. Cell 120, 483–495. 10.1016/j.cell.2005.02.001 15734681

[B5] Baldridge G. D. Kurtti T. J. Munderloh U. G. (2005). Susceptibility of rickettsia monacensis and *Rickettsia peacockii* to cecropin a, ceratotoxin a, and lysozyme. Curr. Microbiol. 51, 233–238. 10.1007/s00284-005-4532-7 16132458

[B6] Ballard J. W. O. (2005). *Drosophila simulans* as a novel model for studying mitochondrial metabolism and aging. Exp. Gerontol. 40, 763–773. 10.1016/j.exger.2005.07.014 16169180

[B7] Ballard J. W. O. Melvin R. G. (2007). Tetracycline treatment influences mitochondrial metabolism and mtDNA density two generations after treatment in *Drosophila*. Insect. Mol. Biol. 16, 799–802. 10.1111/j.1365-2583.2007.00760.x 18093008

[B8] Barnham K. J. Masters C. L. Bush A. I. (2004). Neurodegenerative diseases and oxidative stress. Nat. Rev. Drug Discov. 3, 205–214. 10.1038/nrd1330 15031734

[B9] Bedard K. Lardy B. Krause K.-H. (2007). NOX family NADPH oxidases: not just in mammals. Biochimie 89, 1107–1112. 10.1016/j.biochi.2007.01.012 17400358

[B10] Bergin D. Reeves E. P. Renwick J. Wientjes F. B. Kavanagh K. (2005). Superoxide production in Galleria mellonella hemocytes: identification of proteins homologous to the NADPH oxidase complex of human neutrophils. Infect. Immun. 73, 4161–4170. 10.1128/IAI.73.7.4161-4170.2005 15972506 PMC1168619

[B11] Bian G. Joshi D. Dong Y. Lu P. Zhou G. Pan X. (2013). *Wolbachia* invades *Anopheles stephensi* populations and induces refractoriness to *Plasmodium* infection. Science 340, 748–751. 10.1126/science.1236192 23661760

[B12] Bian G. Xu Y. Lu P. Xie Y. Xi Z. (2010). The endosymbiotic bacterium *Wolbachia* induces resistance to dengue virus in *Aedes aegypti*. PLoS Pathog. 6:e1000833. 10.1371/journal.ppat.1000833 20368968 PMC2848556

[B13] Bonnay F. Cohen-Berros E. Hoffmann M. Kim S. Y. Boulianne G. L. Hoffmann J. A. (2013). big bang gene modulates gut immune tolerance in *Drosophila*. Proc. Natl Acad. Sci. U.S.A. 110, 2957–2962. 10.1073/pnas.1221910110 23378635 PMC3581892

[B14] Bosco-Drayon V. Poidevin M. Boneca I. G. Narbonne-Reveau K. Royet J. Charroux B. (2012). Peptidoglycan sensing by the receptor PGRP-LE in the *Drosophila* gut induces immune responses to infectious bacteria and tolerance to microbiota. Cell Host Microbe 12, 153–165. 10.1016/j.chom.2012.06.002 22901536

[B15] Bourtzis K. Pettigrew M. M. O’Neill S. L. (2000). *Wolbachia* neither induces nor suppresses transcripts encoding antimicrobial peptides. Insect Mol. Biol. 9, 635–639. 10.1046/j.1365-2583.2000.00224.x 11122472

[B16] Brennan C. A. Anderson K. V. (2004). *Drosophila*: the genetics of innate immune recognition and response. Annu. Rev. Immunol. 22, 457–483. 10.1146/annurev.immunol.22.012703.104626 15032585

[B17] Brennan L. J. Haukedal J. A. Earle J. C. Keddie B. Harris H. L. (2012). Disruption of redox homeostasis leads to oxidative DNA damage in spermatocytes of *Wolbachia*-infected *Drosophila simulans*. Insect Mol. Biol. 21, 510–520. 10.1111/j.1365-2583.2012.01155.x 22831171

[B18] Brennan L. J. Keddie B. A. Braig H. R. Harris H. L. (2008). The endosymbiont *Wolbachia pipientis* induces the expression of host antioxidant proteins in an *Aedes albopictus* cell line. PLoS ONE 3:e2083. 10.1371/journal.pone.0002083 18461124 PMC2324199

[B19] Brown D. I. Griendling K. K. (2009). Nox proteins in signal transduction. Free Radic. Biol. Med. 47, 1239–1253. 10.1016/j.freeradbiomed.2009.07.023 19628035 PMC2763943

[B20] Buchon N. Broderick N. A. Lemaitre B. (2013). Gut homeostasis in a microbial world: insights from *Drosophila melanogaster*. Nat. Rev. Microbiol. 11, 615–626. 10.1038/nrmicro3074 23893105

[B21] Buchon N. Silverman N. Cherry S. (2014). Immunity in *Drosophila melanogaster—*from microbial recognition to whole-organism physiology. Nat. Rev. Immunol. 14, 796–810. 10.1038/nri3763 25421701 PMC6190593

[B22] Calabrese E. J. Baldwin L. A. (2002). Defining hormesis. Hum. Exp. Toxicol. 21, 91–97. 10.1191/0960327102ht217oa 12102503

[B23] Carrondo M. A. (2003). Ferritins, iron uptake and storage from the bacterioferritin viewpoint. EMBO J. 22, 1959–1968. 10.1093/emboj/cdg215 12727864 PMC156087

[B24] Carter V. Hurd H. (2010). Choosing anti-*Plasmodium* molecules for genetically modifying mosquitoes: focus on peptides. Trends Parasitol. 26, 582–590. 10.1016/j.pt.2010.07.005 20800543

[B25] Cassat J. E. Skaar E. P. (2013). Iron in infection and immunity. Cell Host Microbe 13, 509–519. 10.1016/j.chom.2013.04.010 23684303 PMC3676888

[B26] Chakrabarti S. Poidevin M. Lemaitre B. (2014). The *Drosophila* MAPK p38c regulates oxidative stress and lipid homeostasis in the intestine. PLoS Genet. 10:e1004659. 10.1371/journal.pgen.1004659 25254641 PMC4177744

[B27] Chávez V. Mohri-Shiomi A. Maadani A. Vega L. A. Garsin D. A. (2007). Oxidative stress enzymes are required for DAF-16-mediated immunity due to generation of reactive oxygen species by *Caenorhabditis elegans*. Genetics 176, 1567–1577. 10.1534/genetics.107.072587 17483415 PMC1931534

[B28] Cheng Q. Ruel T. D. Zhou W. Moloo S. K. Majiwa P. O’Neill S. L. (2000). Tissue distribution and prevalence of *Wolbachia* infections in tsetse flies, *Glossina* spp. Med. Vet. Entomol. 14, 44–50. 10.1046/j.1365-2915.2000.00202.x 10759311

[B29] Chevalier F. Herbinière-Gaboreau J. Charif D. Mitta G. Gavory F. Wincker P. (2012). Feminizing *Wolbachia*: a transcriptomics approach with insights on the immune response genes in *Armadillidium vulgare*. BMC Microbiol. 12:S1. 10.1186/1471-2180-12-S1-S1 22375708 PMC3287506

[B30] Choy A. Roy C. R. (2013). Autophagy and bacterial infection: an evolving arms race. Trends Microbiol. 21, 451–456. 10.1016/j.tim.2013.06.009 23880062 PMC3839292

[B31] Chrostek E. Marialva M. S. P. Esteves S. S. Weinert L. A. Martinez J. Jiggins F. M. (2013). *Wolbachia* variants induce differential protection to viruses in *Drosophila melanogaster*: a phenotypic and phylogenomic analysis. PLoS Genet. 9:e1003896. 10.1371/journal.pgen.1003896 24348259 PMC3861217

[B32] Chrostek E. Marialva M. S. P. Yamada R. O’Neill S. L. Teixeira L. (2014). High anti-viral protection without immune upregulation after interspecies *Wolbachia* transfer. PLoS ONE 9:e99025. 10.1371/journal.pone.0099025 24911519 PMC4049622

[B33] Cirimotich C. M. Dong Y. Clayton A. M. Sandiford S. L. Souza-Neto J. A. Mulenga M. (2011). Natural microbe-mediated refractoriness to *Plasmodium* infection in *Anopheles gambiae*. Science 332, 855–858. 10.1126/science.1201618 21566196 PMC4154605

[B34] Dantoft W. Davis M. M. Lindvall J. M. Tang X. Uvell H. Junell A. (2013). The Oct1 homolog Nubbin is a repressor of NF-κB-dependent immune gene expression that increases the tolerance to gut microbiota. BMC Biol. 11:99. 10.1186/1741-7007-11-99 24010524 PMC3849502

[B35] D’Autréaux B. Toledano M. B. (2007). ROS as signalling molecules: mechanisms that generate specificity in ROS homeostasis. Nat. Rev. Mol. Cell Biol. 8, 813–824. 10.1038/nrm2256 17848967

[B36] De Haes W. Frooninckx L. Van Assche R. Smolders A. Depuydt G. Billen J. (2014). Metformin promotes lifespan through mitohormesis via the peroxiredoxin PRDX-2. Proc. Natl Acad. Sci. U.S.A. 111, E2501–E2509. 10.1073/pnas.1321776111 24889636 PMC4066537

[B37] DeJong R. J. Miller L. M. Molina-Cruz A. Gupta L. Kumar S. Barillas-Mury C. (2007). Reactive oxygen species detoxification by catalase is a major determinant of fecundity in the mosquito *Anopheles gambiae*. Proc. Natl Acad. Sci. U.S.A. 103, 2121–2126. 10.1073/pnas.0608407104 17284604 PMC1892935

[B38] Deretic V. (2010). Autophagy in infection. Curr. Opin. Cell Biol. 22, 252–262. 10.1016/j.ceb.2009.12.009 20116986 PMC2866841

[B39] Deretic V. Saitoh T. Akira S. (2013). Autophagy in infection, inflammation and immunity. Nat. Rev. Immunol. 13, 722–737. 10.1038/nri3532 24064518 PMC5340150

[B40] Diaz-Albiter H. Sant’Anna M. R. V. Genta F. A. Dillon R. J. (2012). Reactive oxygen species-mediated immunity against *Leishmania mexicana* and *Serratia marcescens* in the phlebotomine sand fly *Lutzomyia longipalpis*. J. Biol. Chem. 287, 23995–24003. 10.1074/jbc.M112.376095 22645126 PMC3390674

[B41] Dixon S. J. Stockwell B. R. (2014). The role of iron and reactive oxygen species in cell death. Nature Chem. Biol. 10, 9–17. 10.1038/nchembio.1416 24346035

[B42] Dobson S. L. Bourtzis K. Braig H. R. Jones B. F. Zhou W. Rousset F. (1999). *Wolbachia* infections are distributed throughout insect somatic and germ line tissues. Insect Biochem. Mol. Biol. 29, 153–160. 10196738 10.1016/s0965-1748(98)00119-2

[B43] Dowling D. K. Simmons L. W. (2009). Reactive oxygen species as universal constraints in life-history evolution. Proc. R. Soc. B 276, 1737–1745. 10.1098/rspb.2008.1791 19324792 PMC2674489

[B44] Driver C. Georgiou A. Georgiou G. (2004). The contribution by mitochondrially induced oxidative damage to aging in *Drosophila melanogaster*. Biogerontology 5, 185–192. 10.1023/B:BGEN.0000031156.75376.e3 15190188

[B45] Dunning Hotopp J. C. Lin M. Madupu R. Crabtree J. Angiuoli S. V. Eisen J. (2006). Comparative genomics of emerging human ehrlichiosis agents. PLoS Genet. 2:e21. 10.1371/journal.pgen.0020021 16482227 PMC1366493

[B46] Engel P. Moran N. (2013). The gut microbiota of insects—diversity in structure and function. FEMS Microbiol. Rev. 37, 699–735. 10.1111/1574-6976.12025 23692388

[B47] Erkosar B. Storelli G. Defaye A. Leulier F. (2013). Host-intestinal microbiota mutualism: “learning on the fly”. Cell Host Microbe 13, 8–14. 10.1016/j.chom.2012.12.004 23332152

[B48] Esterházy D. King M. S. Yakovlev G. Hirst J. (2008). Production of reactive oxygen species by complex I (NADH: ubiquinone oxidoreductase) from *Escherichia coli* and comparison to the enzyme from mitochondria. Biochemistry 47, 3964–3971. 10.1021/bi702243b 18307315

[B49] Fauvarque M.-O. Williams M. J. (2011). *Drosophila* cellular immunity: a story of migration and adhesion. J. Cell Sci. 124, 1373–1382. 10.1242/jcs.064592 21502134

[B50] Ferrandon D. Imler J.-L. Hetru C. Hoffmann J. A. (2007). The *Drosophila* systemic immune response: sensing and signalling during bacterial and fungal infections. Nat. Rev. Immunol. 7, 862–874. 10.1038/nri2194 17948019

[B51] Ferreira Á. G. Naylor H. Esteves S. S. Pais I. S. Martins N. E. Teixeira L. (2014). The Toll-Dorsal pathway is required for resistance to viral oral infection in *Drosophila*. PLoS Pathog. 10:e1004507. 10.1371/journal.ppat.1004507 25473839 PMC4256459

[B52] Finkel T. (2011). Signal transduction by reactive oxygen species. J. Cell Biol. 194, 7–15. 10.1083/jcb.201102095 21746850 PMC3135394

[B53] Flora G. Gupta D. Tiwari A. (2012). Toxicity of lead: a review with recent updates. Interdiscip. Toxicol. 5, 47–58. 10.2478/v10102-012-0009-2 23118587 PMC3485653

[B54] Forbes V. E. (2000). Is hormesis an evolutionary expectation? Funct. Ecol. 14, 12–24. 10.1046/j.1365-2435.2000.00392.x

[B55] Frost C. L. Pollock S. W. Smith J. E. Hughes W. O. H. (2014). *Wolbachia* in the flesh: symbiont intensities in germ-line and somatic tissues challenge the conventional view of *Wolbachia* transmission routes. PLoS ONE 9:e95122. 10.1371/journal.pone.0095122 24988478 PMC4079706

[B56] García-Gil de Muñoz F. Lanz-Mendoza H. Hernández-Hernández F. C. (2007). Free radical generation during the activation of hemolymph prepared from the homopteran *Dactylopius coccus*. Arch. Insect Biochem. Physiol. 65, 20–28. 10.1002/arch.20174 17427930

[B57] Gems D. Partridge L. (2008). Stress-response hormesis and aging: “that which does not kill us makes us stronger”. Cell Metab. 7, 200–203. 10.1016/j.cmet.2008.01.001 18316025

[B58] Gerschman R. Gilbert D. L. Nye S. W. Dwyer P. Fenn W. O. (1954). Oxygen poisoning and X-irradiation: a mechanism in common. Science 119, 623–626. 13156638 10.1126/science.119.3097.623

[B59] Gill A. C. Darby A. C. Makepeace B. L. (2014). Iron necessity: the secret of *Wolbachia*’s success? PLoS Negl. Trop. Dis. 8:e3224. 10.1371/journal.pntd.0003224 25329055 PMC4199550

[B60] Gonçalves R. L. S. Oliveira J. H. M. Oliveira G. A. Andersen J. F. Oliveira M. F. Oliveira P. L. (2012). Mitochondrial reactive oxygen species modulate mosquito susceptibility to *Plasmodium* infection. PLoS ONE 7:e41083. 10.1371/journal.pone.0041083 22815925 PMC3399787

[B61] Graça-Souza A. V. Maya-Monteiro C. Paiva-Silva G. O. Braz G. R. C. Paes M. C. Sorgine M. H. F. (2006). Adaptations against heme toxicity in blood-feeding arthropods. Insect Biochem. Mol. Biol. 36, 322–335. 10.1016/j.ibmb.2006.01.009 16551546

[B62] Griendling K. K. FitzGerald G. A. (2003). Oxidative stress and cardiovascular injury. Part I: basic mechanisms and in vivo monitoring of ROS. Circulation 108, 1912–1916. 10.1161/01.CIR.0000093660.86242.BB 14568884

[B63] Ha E.-M. Lee K.-A. Park S. H. Kim S.-H. Nam H.-J. Lee H.-Y. (2009a). Regulation of DUOX by the Gαq-phospholipase Cβ–Ca^2+^ pathway in *Drosophila* gut immunity. Dev. Cell 16, 386–397. 10.1016/j.devcel.2008.12.015 19289084

[B64] Ha E.-M. Lee K.-A. Seo Y. Y. Kim S.-H. Lim J.-H. Oh B.-H. (2009b). Coordination of multiple dual oxidase-regulatory pathways in responses to commensal and infectious microbes in *Drosophila* gut. Nat. Immunol. 10, 949–957. 10.1038/ni.1765 19668222

[B65] Ha E.-M. Oh C.-T. Bae Y. S. Lee W.-J. (2005a). A direct role for dual oxidase in *Drosophila* gut immunity. Science 310, 847–850. 10.1126/science.1117311 16272120

[B66] Ha E.-M. Oh C.-T. Ryu J.-H. Bae Y.-S. Kang S.-W. Jang I.-H. (2005b). An antioxidant system required for host protection against gut infection in *Drosophila*. Dev. Cell 8, 125–132. 10.1016/j.devcel.2004.11.007 15621536

[B67] Hamanaka R. B. Chandel N. S. (2010). Mitochondrial reactive oxygen species regulate cellular signaling and dictate biological outcomes. Trends Biochem. Sci. 35, 505–513. 10.1016/j.tibs.2010.04.002 20430626 PMC2933303

[B68] Harman D. (1956). Aging: a theory based on free radical and radiation chemistry. J. Gerontol. 11, 298–300. 13332224 10.1093/geronj/11.3.298

[B69] Harman D. (1972). The biologic clock: the mitochondria? J. Am. Geriatr. Soc. 20, 145–147. 5016631 10.1111/j.1532-5415.1972.tb00787.x

[B70] Haukedal J. A. (2013). Regulation of Wolbachia Density Within Drosophila Simulans. Ph.D. thesis, University of Alberta, Edmonton, Alberta.

[B71] Hedges L. M. Brownlie J. C. O’Neill S. L. Johnson K. N. (2008). *Wolbachia* and virus protection in insects. Science 322, 702. 10.1126/science.1162418 18974344

[B72] Hetru C. Hoffmann J. A. (2009). NF-κB in the immune response of *Drosophila*. Cold Spring Harb. Perspect. Biol. 1:a000232. 10.1101/cshperspect.a000232 20457557 PMC2882123

[B73] Hilgenboecker K. Hammerstein P. Schlattmann P. Telschow A. Werren J. H. (2008). How many species are infected with *Wolbachia*?—a statistical analysis of current data. FEMS Microbiol. Lett. 281, 215–220. 10.1111/j.1574-6968.2008.01110.x 18312577 PMC2327208

[B74] Ho Y.-S. Magnenat J.-L. Gargano M. Cao J. (1998). The nature of antioxidant defense mechanisms: a lesson from transgenic studies. Environ. Health Perspect. 106, 1219–1228. 9788901 10.1289/ehp.98106s51219 PMC1533365

[B75] Holmström K. M. Finkel T. (2014). Cellular mechanisms and physiological consequences of redox-dependent signalling. Nat. Rev. Mol. Cell Biol. 15, 411–421. 10.1038/nrm3801 24854789

[B76] Hornett E. A. Charlat S. Duplouy A. M. R. Davies N. Roderick G. K. Wedell N. (2006). Evolution of male-killer suppression in a natural population. PLoS Biol. 4:e283. 10.1371/journal.pbio.0040283 16933972 PMC1551922

[B77] Huang J. Canadien V. Lam G. Y. Steinberg B. E. Dinauer M. C. Magalhaes M. A. O. (2009). Activation of antibacterial autophagy by NADPH oxidases. Proc. Natl Acad. Sci. U.S.A. 106, 6226–6231. 10.1073/pnas.0811045106 19339495 PMC2664152

[B78] Hughes G. L. Ren X. Ramirez J. L. Sakamoto J. M. Bailey J. A. Jedlicka A. E. (2011). *Wolbachia* infections in *Anopheles gambiae* cells: transcriptomic characterization of a novel host-smbiont interaction. PLoS Pathog. 7:e1001296. 10.1371/journal.ppat.1001296 21379333 PMC3040664

[B79] Huigens M. E. (2003). On the Evolution of Wolbachia-induced Parthenogenesis in Trichogramma Wasps. Ph.D. thesis, Wageningen University, Wageningen, Netherlands.

[B80] Hurst G. D. D. Anbutsu H. Kutsukake M. Fukatsu T. (2003). Hidden from the host: *Spiroplasma* bacteria infecting *Drosophila* do not cause an immune response, but are suppressed by ectopic immune activation. Insect Mol. Biol. 12, 93–97. 10.1046/j.1365-2583.2003.00380.x 12542640

[B81] Hurst G. D. D. Jiggins F. M. (2005). Problems with mitochondrial DNA as a marker in population, phylogeographic and phylogenetic studies: the effects of inherited symbionts. Proc. R. Soc. B 272, 1525–1534. 10.1098/rspb.2005.3056 16048766 PMC1559843

[B82] Hutchence K. J. Fischer B. Paterson S. Hurst G. D. D. (2011). How do insects react to novel inherited symbionts? A microarray analysis of *Drosophila melanogaster* response to the presence of natural and introduced *Spiroplasma*. Mol. Ecol. 20, 950–958. 10.1111/j.1365-294X.2010.04974.x 21255169

[B83] Ijichi N. Kondo N. Matsumoto R. Shimada M. Ishikawa H. Fukatsu T. (2002). Internal spatiotemporal population dynamics of infection with three *Wolbachia* strains in the adzuki bean beetle, *Callosobruchus chinensis* (*Coleoptera: Bruchidae*). Appl. Environ. Microbiol. 68, 4074–4080. 10.1128/AEM.68.8.4074-4080.2002 12147509 PMC124025

[B84] Isaksson C. Sheldon B. C. Uller T. (2011). The challenges of integrating oxidative stress into life-history biology. Bioscience 61, 194–202. 10.1525/bio.2011.61.3.5

[B85] Ishii K. Hamamoto H. Kamimura M. Sekimizu K. (2008). Activation of the silkworm cytokine by bacterial and fungal cell wall components via a reactive oxygen species-triggered mechanism. J. Biol. Chem. 283, 2185–2191. 10.1074/jbc.M705480200 17947232

[B86] Jeney V. Balla J. Yachie A. Varga Z. Vercellotti G. M. Eaton J. W. (2002). Pro-oxidant and cytotoxic effects of circulating heme. Blood 100, 879–887. 10.1182/blood.V100.3.879 12130498

[B87] Jiravanichpaisal P. Lee B. L. Söderhäll K. (2006). Cell-mediated immunity in arthropods: hematopoiesis, coagulation, melanization and opsonization. Immunobiology 211, 213–236. 10.1016/j.imbio.2005.10.015 16697916

[B88] Johnson K. N. (2015). Bacteria and antiviral immunity in insects. Curr. Opin. Insect Sci. 8, 97–103. 10.1016/j.cois.2015.01.008 32846693

[B89] Kambris Z. Blagborough A. M. Pinto S. B. Blagrove M. S. C. Godfray H. C. J. Sinden R. E. (2010). *Wolbachia* stimulates immune gene expression and inhibits *Plasmodium* development in *Anopheles gambiae*. PLoS Pathog. 6: e1001143. 10.1371/journal.ppat.1001143 20949079 PMC2951381

[B90] Kambris Z. Cook P. E. Phuc H. K. Sinkins S. P. (2009). Immune activation by life-shortening *Wolbachia* and reduced filarial competence in mosquitoes. Science 326, 134–136. 10.1126/science.1177531 19797660 PMC2867033

[B91] Kaneko T. Yano T. Aggarwal K. Lim J.-H. Ueda K. Oshima Y. (2006). PGRP-LC and PGRP-LE have essential yet distinct functions in the *Drosophila* immune response to monomeric DAP-type peptidoglycan. Nat. Immunol. 7, 715–723. 10.1038/ni1356 16767093

[B92] Kharade S. V. Mittal N. Das S. P. Sinha P. Roy N. (2005). Mrg19 depletion increases *S. cerevisiae* lifespan by augmenting ROS defence. FEBS Lett. 579, 6809–6813. 10.1016/j.febslet.2005.11.017 16336970

[B93] Khush R. S. Leulier F. Lemaitre B. (2001). *Drosophila* immunity: two paths to NF-κB. Trends Immunol. 22, 260–264. 10.1016/S1471-4906(01)01887-7 11323284

[B94] Kim S.-H. Lee W.-J. (2014). Role of DUOX in gut inflammation: lessons from *Drosophila* model of gut-microbiota interactions. Front. Cell Infect. Microbiol. 3:116. 10.3389/fcimb.2013.00116 24455491 PMC3887270

[B95] Kirkwood T. B. L. Kowald A. (2012). The free-radical theory of ageing—older, wiser and still alive. Bioessays 34, 692–700. 10.1002/bies.201200014 22641614

[B96] Koehncke A. Telschow A. Werren J. H. Hammerstein P. (2009). Life and death of an influential passenger: *Wolbachia* and the evolution of CI-modifiers by their hosts. PLoS ONE 4:e4425. 10.1371/journal.pone.0004425 19209229 PMC2635967

[B97] Kremer N. Charif D. Henri H. Gavory F. Wincker P. Mavingui P. (2012). Influence of *Wolbachia* on host gene expression in an obligatory symbiosis. BMC Microbiol. 12:S7. 10.1186/1471-2180-12-S1-S7 22376153 PMC3287518

[B98] Kremer N. Dedeine F. Charif D. Finet C. Allemand R. Vavre F. (2010). Do variable compensatory mechanisms explain the polymorphism of the dependence phenotype in the *Asobara* tabida-*Wolbachia* association? Evolution 64, 2969–2979. 10.1111/j.1558-5646.2010.01034.x 20482609

[B99] Kremer N. Voronin D. Charif D. Mavingui P. Mollereau B. Vavre F. (2009). *Wolbachia* interferes with ferritin expression and iron metabolism in insects. PLoS Pathog. 5: e1000630. 10.1371/journal.ppat.1000630 19851452 PMC2759286

[B100] Kumar S. Christophides G. K. Cantera R. Charles B. Han Y. S. Meister S. (2003). The role of reactive oxygen species on *Plasmodium* melanotic encapsulation in *Anopheles gambiae*. Proc. Natl Acad. Sci. U.S.A. 100, 14139–14144. 10.1073/pnas.2036262100 14623973 PMC283559

[B101] Kuraishi T. Hori A. Kurata S. (2013). Host-microbe interactions in the gut of *Drosophila melanogaster*. Front. Physiol. 4:375. 10.3389/fphys.2013.00375 24381562 PMC3865371

[B102] Kurata S. (2010). Extracellular and intracellular pathogen recognition by *Drosophila* PGRP-LE and PGRP-LC. Int. Immunol. 22, 143–148. 10.1093/intimm/dxp128 20089584 PMC2829096

[B103] Kurz M. Iturbe-Ormaetxe I. Jarrott R. Shouldice S. R. Wouters M. A. Frei P. (2009). Structural and functional characterization of the oxidoreductase a-DsbA1 from *Wolbachia pipientis*. Antioxid. Redox Signal. 11, 1485–1500. 10.1089/ars.2008.2420 19265485

[B104] Lambeth J. D. (2004). Nox enzymes and the biology of reactive oxygen. Nat. Rev. Immunol. 4, 181–189. 10.1038/nri1312 15039755

[B105] Lambeth J. D. Neish A. S. (2014). Nox enzymes and new thinking on reactive oxygen: a double-edged sword revisited. Annu. Rev. Pathol. Mech. Dis. 9, 119–145. 10.1146/annurev-pathol-012513-104651 24050626

[B106] Lamiable O. Imler J.-L. (2014). Induced antiviral innate immunity in *Drosophila*. Curr. Opin. Microbiol. 20, 62–68. 10.1016/j.mib.2014.05.006 24907422 PMC4133299

[B107] Landmann F. Orsi G. A. Loppin B. Sullivan W. J. (2009). *Wolbachia* -mediated cytoplasmic incompatibility is associated with impaired histone deposition in the male pronucleus. PLoS Pathog. 5: e1000343. 10.1371/journal.ppat.1000343 19300496 PMC2652114

[B108] Lapointe J. Hekimi S. (2010). When a theory of aging ages badly. Cell. Mol. Life Sci. 67, 1–8. 10.1007/s00018-009-0138-8 19730800 PMC4053417

[B109] Le Clec’h W. Braquart-Varnier C. Raimond M. Ferdy J.-B. Bouchon D. Sicard M. (2012). High virulence of *Wolbachia* after host switching: when autophagy hurts. PLoS Pathog. 8:e1002844. 10.1371/journal.ppat.1002844 22876183 PMC3410869

[B110] Lee K.-A Kim B. Bhin J. Kim D. H. You H. Kim E.-K (2015). Bacterial uracil modulates *Drosophila* DUOX-dependent gut immunity via *Hedgehog*-induced signaling endosomes. Cell Host Microbe 17, 191–204. 10.1016/j.chom.2014.12.012 25639794

[B111] Lee K.-A. Kim S.-H. Kim E.-K. Ha E.-M. You H. Kim B. (2013). Bacterial-derived uracil as a modulator of mucosal immunity and gut–microbe homeostasis in *Drosophila*. Cell 153, 797–811. 10.1016/j.cell.2013.04.009 23663779

[B112] Lee W.-J. Brey P. T. (2013). How microbiomes influence metazoan development: insights from history and *Drosophila* modeling of gut-microbe interactions. Annu. Rev. Cell Dev. Biol. 29, 571–592. 10.1146/annurev-cellbio-101512-122333 23808845

[B113] Lemaitre B. Hoffmann J. (2007). The host defense of *Drosophila melanogaster*. Annu. Rev. Immunol. 25, 697–743. 10.1146/annurev.immunol.25.022106.141615 17201680

[B114] Leulier F. Parquet C. Pili-Floury S. Ryu J. H. Caroff M. Lee W.-J. (2003). The *Drosophila* immune system detects bacteria through specific peptidoglycan recognition. Nat. Immunol. 4, 478–484. 10.1038/ni922 12692550

[B115] Lhocine N. Ribeiro P. S. Buchon N. Wepf A. Wilson R. Tenev T. (2008). PIMS modulates immune tolerance by negatively regulating *Drosophila* innate immune signaling. Cell Host Microbe 4, 147–158. 10.1016/j.chom.2008.07.004 18692774

[B116] Liu H. Bao W. Lin M. Niu H. Rikihisa Y. (2012a). Ehrlichia type IV secretion effector ECH0825 is translocated to mitochondria and curbs ROS and apoptosis by upregulating host MnSOD. Cell. Microbiol. 14, 1037–1050. 10.1111/j.1462-5822.2012.01775.x 22348527 PMC3371182

[B117] Liu L. Dai J. Zhao Y. O. Narasimhan S. Yang Y. Zhang L. (2012b). Ixodes scapularis JAK-STAT pathway regulates tick antimicrobial peptides, thereby controlling the agent of human granulocytic anaplasmosis. J. Infect. Dis. 206, 1233–1241. 10.1093/infdis/jis484 22859824 PMC3448968

[B118] Login F. H. Balmand S. Vallier A. Vincent-Monégat C. Vigneron A. Weiss-Gayet M. (2011). Antimicrobial peptides keep insect endosymbionts under control. Science 334, 362–365. 10.1126/science.1209728 22021855

[B119] Louis C. Nigro L. (1989). Ultrastructural evidence of *Wolbachia* Rickettsiales in *Drosophila simulans* and their relationships with unidirectional cross-incompatibility. J. Invertebr. Pathol. 54, 39–44. 10.1016/0022-2011(89)90137-7

[B120] Lundgren J. G. Jurat-Fuentes J. L. (2012). “Physiology and ecology of host defense against microbial invaders,” in Insect Pathology, Second Edition, eds Vega F. E. Kaya H. K. (San Diego, CA: Academic Press), 461–480.

[B121] Maritim A. C. Sanders R. A. Watkins J. B. III (2003). Diabetes, oxidative stress, and antioxidants: a review. J. Biochem. Mol. Toxicol. 17, 24–38. 10.1002/jbt.10058 12616644

[B122] Martinez J. Longdon B. Bauer S. Chan Y.-S. Miller W. J. Bourtzis K. (2014). Symbionts commonly provide broad spectrum resistance to viruses in insects: a comparative analysis of *Wolbachia* strains. PLoS Pathog. 10:e1004369. 10.1371/journal.ppat.1004369 25233341 PMC4169468

[B123] Martinez J. Ok S. Smith S. Snoeck K. Day J. P. Jiggins F. M. (2015). Should symbionts be nice or selfish? Antiviral effects of *Wolbachia* are costly but reproductive parasitism is not. PLoS Pathog. 11:e1005021. 10.1371/journal.ppat.1005021 26132467 PMC4488530

[B124] Masri L. Cremer S. (2014). Individual and social immunisation in insects. Trends Immunol. 35, 471–482. 10.1016/j.it.2014.08.005 25245882

[B125] Mateos M. Castrezana S. J. Nankivell B. J. Estes A. M. Markow T. A. Moran N. A. (2006). Heritable endosymbionts of *Drosophila*. Genetics 174, 363–376. 10.1534/genetics.106.058818 16783009 PMC1569794

[B126] McClure C. D. Zhong W. Hunt V. L. Chapman F. M. Hill F. V. Priest N. K. (2014). Hormesis results in trade-offs with immunity. Evolution 68, 2225–2233. 10.1111/evo.12453 24862588 PMC4282086

[B127] McCord J. M. Fridovich I. (1969). Superoxide dismutase. An enzymic function for erythrocuprein (hemocuprein). J. Biol. Chem. 244, 6049–6055. 5389100

[B128] McCord J. M. Keele B. B. Jr Fridovich I. (1971). An enzyme-based theory of obligate anaerobiosis: the physiological function of superoxide dismutase. Proc. Natl Acad. Sci. U.S.A. 68, 1024–1027. 10.1073/pnas.68.5.1024 4995818 PMC389105

[B129] McKean K. A. Lazzaro B. (2011). “The costs of immunity and the evolution of immunological defense mechanisms,” in Mechanisms of Life History Evolution, eds Flatt T. Heyland A. (Oxford: Oxford University Press), 299–310.

[B130] Merkling S. H. van Rij R. P. (2013). Beyond RNAi: antiviral defense strategies in *Drosophila* and mosquito. J. Insect Physiol. 59, 159–170. 10.1016/j.jinsphys.2012.07.004 22824741

[B131] Messens J. Rouhier N. Collet J.-F. (2013). “Redox Homeostasis,” in Oxidative Stress and Redox Regulation, eds Jakob U. Reichmann D. (Dordrecht: Springer), 59–84.

[B132] Metcalfe N. B. Alonso-Alvarez C. (2010). Oxidative stress as a life-history constraint: the role of reactive oxygen species in shaping phenotypes from conception to death. Funct. Ecol. 24, 984–996. 10.1111/j.1365-2435.2010.01750.x

[B133] Mikonranta L. Mappes J. Kaukoniitty M. Freitak D. (2014). Insect immunity: oral exposure to a bacterial pathogen elicits free radical response and protects from a recurring infection. Front. Zool. 11:23. 10.1186/1742-9994-11-23 24602309 PMC3975449

[B134] Molina-Cruz A. DeJong R. J. Charles B. Gupta L. Kumar S. Jaramillo-Gutierrez G. (2008). Reactive oxygen species modulate *Anopheles gambiae* immunity against bacteria and *Plasmodium*. J. Biol. Chem. 283, 3217–3223. 10.1074/jbc.M705873200 18065421

[B135] Molloy J. C. Sinkins S. P. (2015). *Wolbachia* do not induce reactive oxygen species-dependent immune pathway activation in *Aedes albopictus*. Viruses 7, 4624–4639. 10.3390/v7082836 26287231 PMC4576197

[B136] Monaghan P. Metcalfe N. B. Torres R. (2009). Oxidative stress as a mediator of life history trade-offs: mechanisms, measurements and interpretation. Ecol. Lett. 12, 75–92. 10.1111/j.1461-0248.2008.01258.x 19016828

[B137] Moné Y. Monnin D. Kremer N. (2014). The oxidative environment: a mediator of interspecies communication that drives symbiosis evolution. Proc. R. Soc. B 281, 20133112. 10.1098/rspb.2013.3112 24807248 PMC4024279

[B138] Moreira L. A. Iturbe-Ormaetxe I. Jeffery J. A. Lu G. Pyke A. T. Hedges L. M. (2009). A *Wolbachia* symbiont in *Aedes aegypti* limits infection with Dengue, Chikungunya, and *Plasmodium*. Cell 139, 1268–1278. 10.1016/j.cell.2009.11.042 20064373

[B139] Mouchiroud L. Houtkopper R. H. Moullan N. Katsyuba E. Ryu D. Cantó C. (2013). The NAD+/sirtuin pathway modulates longevity through activation of mitochondrial UPR and FOXO signaling. Cell 154, 430–441. 10.1016/j.cell.2013.06.016 23870130 PMC3753670

[B140] Nairz M. Haschka D. Demetz E. Weiss G. (2014). Iron at the interface of immunity and infection. Front. Pharmacol. 5:152. 10.3389/fphar.2014.00152 25076907 PMC4100575

[B141] Nakamoto M. Moy R. H. Xu J. Bambina S. Yasunaga A. Shelly S. S. (2012). Virus recognition by Toll-7 activates antiviral autophagy in *Drosophila*. Immunity 36, 658–667. 10.1016/j.immuni.2012.03.003 22464169 PMC3334418

[B142] Nappi A. J. Vass E. (1998). Hydrogen peroxide production in immune-reactive *Drosophila melanogaster*. J. Parasitol. 84, 1150–1157. 9920305

[B143] Nappi A. J. Vass E. (2002). Interactions of iron with reactive intermediates of oxygen and nitrogen. Dev. Neurosci. 24, 134–142. 10.1159/000065697 12401951

[B144] Nappi A. J. Vass E. Frey F. Carton Y. (1995). Superoxide anion generation in *Drosophila* during melanotic encapsulation of parasites. Eur. J. Cell Biol. 68, 450–456. 8690025

[B145] Nathan C. Cunningham-Bussel A. (2013). Beyond oxidative stress: an immunologist’s guide to reactive oxygen species. Nat. Rev. Immunol. 13, 349–361. 10.1038/nri3423 23618831 PMC4250048

[B146] Neyen C. Poidevin M. Roussel A. Lemaitre B. (2012). Tissue- and ligand-specific sensing of Gram-negative infection in *Drosophila* by PGRP-LC isoforms and PGRP-LE. J. Biol. Chem. 189, 1886–1897. 10.4049/jimmunol.1201022 22772451

[B147] Oliveira J. H. M. Gonçalves R. L. S. Lara F. A. Dias F. A. Gandara A. C. P. Menna-Barreto R. F. S. (2011). Blood meal-derived heme decreases ROS levels in the midgut of *Aedes aegypti* and allows proliferation of intestinal microbiota. PLoS Pathog. 7:e1001320. 10.1371/journal.ppat.1001320 21445237 PMC3060171

[B148] Oliveira M. F. Silva J. R. Dansa-Petretski M. de Souza W. Lins U. Braga C. M. S. (1999). Haem detoxification by an insect. Nature 400, 517–518. 10.1038/22910 10448851

[B149] Osborne S. E. Leong Y. S. O’Neill S. L. Johnson K. N. (2009). Variation in antiviral protection mediated by different *Wolbachia* strains in *Drosophila simulans*. PLoS Pathog. 5:e1000656. 10.1371/journal.ppat.1000656 19911047 PMC2768908

[B150] Paiva-Silva G. O. Cruz-Oliveira C. Nakayasu E. S. Maya-Monteiro C. M. Dunkov B. C. Masuda H. (2006). A heme-degradation pathway in a blood-sucking insect. Proc. Natl Acad. Sci. U.S.A. 103, 8030–8035. 10.1073/pnas.0602224103 16698925 PMC1472424

[B151] Pan X. Zhou G. Wu J. Bian G. Lu P. Raikhel A. S. (2012). *Wolbachia* induces reactive oxygen species (ROS)-dependent activation of the Toll pathway to control dengue virus in the mosquito *Aedes aegypti*. Proc. Natl Acad. Sci. U.S.A. 109, E23–E31. 10.1073/pnas.1116932108 22123956 PMC3252928

[B152] Pannebakker B. A. Loppin B. Elemans C. P. H. Humblot L. Vavre F. (2007). Parasitic inhibition of cell death facilitates symbiosis. Proc. Natl Acad. Sci. U.S.A. 104, 213–215. 10.1073/pnas.0607845104 17190825 PMC1765438

[B153] Paredes J. C. Welchman D. P. Poidevin M. Lemaitre B. (2011). Negative regulation by amidase PGRPs shapes the *Drosophila* antibacterial response and protects the fly from innocuous infection. Immunity 35, 770–779. 10.1016/j.immuni.2011.09.018 22118526

[B154] Parkes T. L. Kirby K. Phillips J. P. Hilliker A. J. (1998). Transgenic analysis of the cSOD-null phenotypic syndrome in *Drosophila*. Genome 41, 642–651. 9809435

[B155] Parsons L. M. Lin F. Orban J. (2006). Peptidoglycan recognition by Pal, an outer membrane lipoprotein. Biochemistry 45, 2122–2128. 10.1021/bi052227i 16475801

[B156] Péan C. B. Dionne M. S. (2014). Intracellular infections in *Drosophila melanogaster*: host defense and mechanisms of pathogenesis. Dev. Comp. Immunol. 42, 57–66. 10.1016/j.dci.2013.04.013 23648644

[B157] Pereira L. S. Oliviera P. L. Barja-Fidalgo C. Daffre S. (2001). Production of reactive oxygen species by hemocytes from the cattle tick *Boophilus microplus*. Exp. Parasitol. 99, 66–72. 10.1006/expr.2001.4657 11748959

[B158] Pichon S. Bouchon D. Cordaux R. Chen L. Garrett R. A. Grève P. (2009). Conservation of the type IV secretion system throughout *Wolbachia* evolution. Biochem. Biophys. Res. Commun. 385, 557–562. 10.1016/j.bbrc.2009.05.118 19486895

[B159] Pigeault R. Braquart-Varnier C. Marcadé I. Mappa G. Mottin E. Sicard M. (2014). Modulation of host immunity and reproduction by horizontally acquired *Wolbachia*. J. Insect Physiol. 70, 125–133. 10.1016/j.jinsphys.2014.07.005 25108053

[B160] Raha S. Robinson B. H. (2000). Mitochondria, oxygen free radicals, disease and ageing. Trends Biochem. Sci. 25, 502–508. 10.1016/S0968-0004(00)01674-1 11050436

[B161] Rainey S. M. Shah P. Kohl A. Dietrich I. (2014). Understanding the *Wolbachia*-mediated inhibition of arboviruses in mosquitoes: progress and challenges. J. Gen. Virol. 95, 517–530. 10.1099/vir.0.057422-0 24343914

[B162] Rancès E. Johnson T. K. Popovici J. Iturbe-Ormaetxe I. Zakir T. Warr C. G. (2013). The Toll and Imd pathways are not required for *Wolbachia*-mediated dengue virus interference. J. Virol. 87, 11945–11949. 10.1128/JVI.01522-13 23986574 PMC3807350

[B163] Rancès E. Ye Y. H. Woolfit M. McGraw E. A. O’Neill S. L. (2012). The relative importance of innate immune priming in *Wolbachia*-mediated dengue interference. PLoS Pathog. 8:e1002548. 10.1371/journal.ppat.1002548 22383881 PMC3285598

[B164] Ray P. D. Huang B.-W. Tsuji Y. (2012). Reactive oxygen species (ROS) homeostasis and redox regulation in cellular signaling. Cell. Signal. 24, 981–990. 10.1016/j.cellsig.2012.01.008 22286106 PMC3454471

[B165] Reczek C. R. Chandel N. S. (2015). ROS-dependent signal transduction. Curr. Opin. Cell Biol. 33, 8–13. 10.1016/j.ceb.2014.09.010 25305438 PMC4380867

[B166] Rigaud T. Juchault P. (1992). Genetic control of the vertical transmission of a cytoplasmic sex factor in *Armadillidium vulgare* Latr. (*Crustacea, Oniscidea*). Heredity 68, 47–52.

[B167] Ristow M. Schmeisser K. (2014). Mitohormesis: promoting health and lifespan by increased levels of reactive oxygen species (ROS). Dose Resp. 12, 288–341. 10.2203/dose-response.13-035.Ristow 24910588 PMC4036400

[B168] Rolff J. Reynolds S. E. (2009). Insect Infection and Immunity. Oxford: Oxford University Press.

[B169] Roth O. Sadd B. M. Schmid-Hempel P. Kurtz J. (2009). Strain-specific priming of resistance in the red flour beetle, *Tribolium castaneum*. Proc. R. Soc. B 276, 145–151. 10.1098/rspb.2008.1157 18796392 PMC2614262

[B170] Royer-Pokora B. Kunkel L. M. Monaco A. P. Goff S. C. Newburger P. E. Baehner R. L. (1986). Cloning of the gene for an inherited human disorder—chronic granulomatous disease—on the basis of its chromosomal location. Nature 322, 32–38. 10.1038/322032a0 2425263

[B171] Royet J. Dziarski R. (2007). Peptidoglycan recognition proteins: pleiotropic sensors and effectors of antimicrobial defences. Nat. Rev. Microbiol. 5, 264–277. 10.1038/nrmicro1620 17363965

[B172] Royet J. Gupta D. Dziarski R. (2011). Peptidoglycan recognition proteins: modulators of the microbiome and inflammation. Nat. Rev. Immunol. 11, 837–851. 10.1038/nri3089 22076558

[B173] Ryu J.-H. Ha E.-M. Lee W.-J. (2010). Innate immunity and gut–microbe mutualism in *Drosophila*. Dev. Comp. Immunol. 34, 369–376. 10.1016/j.dci.2009.11.010 19958789

[B174] Ryu J.-H. Ha E.-M. Oh C.-T. Seol J.-H. Brey P. T. Jin I. (2006). An essential complementary role of NF-κB pathway to microbicidal oxidants in *Drosophila* gut immunity. EMBO J. 25, 3693–3701. 10.1038/sj.emboj.7601233 16858400 PMC1538556

[B175] Ryu J.-H. Kim S.-H. Lee H.-Y. Bai J. Y. Nam Y.-D. Bae J.-W. (2008). Innate immune homeostasis by the homeobox gene Caudal and commensal-gut mutualism in *Drosophila*. Science 319, 777–782. 10.1126/science.1149357 18218863

[B176] Sabin L. R. Hanna S. L. Cherry S. (2010). Innate antiviral immunity in *Drosophila*. Curr. Opin. Immunol. 22, 4–9. 10.1016/j.coi.2010.01.007 20137906 PMC2831143

[B177] Sansonetti P. J. Medzhitov R. (2009). Learning tolerance while fighting ignorance. Cell 138, 416–420. 10.1016/j.cell.2009.07.024 19665961

[B178] Scherz-Shouval R. Elazar Z. (2011). Regulation of autophagy by ROS: physiology and pathology. Trends Biochem. Sci. 36, 30–38. 10.1016/j.tibs.2010.07.007 20728362

[B179] Schieber M. Chandel N. S. (2014). ROS function in redox signaling and oxidative stress. Curr. Biol. 24, R453–R462. 10.1016/j.cub.2014.03.034 24845678 PMC4055301

[B180] Schmid-Hempel P. (2003). Variation in immune defence as a question of evolutionary ecology. Proc. R. Soc. B 270, 357–366. 10.1098/rspb.2002.2265 12639314 PMC1691258

[B181] Schneider D. S. Ayres J. S. (2008). Two ways to survive infection: what resistance and tolerance can teach us about treating infectious diseases. Nat. Rev. Immunol. 8, 889–895. 10.1038/nri2432 18927577 PMC4368196

[B182] Schulenburg H. Kurtz J. Moret Y. Siva-Jothy M. T. (2009). Introduction. Ecological immunology. Phil. Trans. R. Soc. B 364, 3–14. 10.1098/rstb.2008.0249 18926970 PMC2666701

[B183] Schulz T. J. Zarse K. Voigt A. Urban N. Birringer M. Ristow M. (2007). Glucose restriction extends *Caenorhabditis elegans* life span by inducing mitochondrial respiration and increasing oxidative stress. Cell Metab. 6, 280–293. 10.1016/j.cmet.2007.08.011 17908557

[B184] Sena L. A. Chandel N. S. (2012). Physiological roles of mitochondrial reactive oxygen species. Mol. Cell 48, 158–167. 10.1016/j.molcel.2012.09.025 23102266 PMC3484374

[B185] Sheldon B. C. Verhulst S. (1996). Ecological immunology: costly parasite defences and trade-offs in evolutionary ecology. Trends Ecol. Evol. 11, 317–321. 10.1016/0169-5347(96)10039-2 21237861

[B186] Simon H.-U. Haj-Yehia A. Levi-Schaffer F. (2000). Role of reactive oxygen species (ROS) in apoptosis induction. Apoptosis 5, 415–418. 10.1023/A:1009616228304 11256882

[B187] Siozios S. Sapountzis P. Ioannidis P. Bourtzis K. (2008). *Wolbachia* symbiosis and insect immune response. Insect Sci. 15, 89–100. 10.1111/j.1744-7917.2008.00189.x

[B188] Sommer F. Bäckhed F. (2013). The gut microbiota—masters of host development and physiology. Nat. Rev. Microbiol. 11, 227–238. 10.1038/nrmicro2974 23435359

[B189] Speakman J. R. Garratt M. (2014). Oxidative stress as a cost of reproduction: beyond the simplistic trade-off model. Bioessays 36, 93–106. 10.1002/bies.201300108 24285005

[B190] Speakman J. R. Selman C. (2011). The free-radical damage theory: accumulating evidence against a simple link of oxidative stress to ageing and lifespan. Bioessays 33, 255–259. 10.1002/bies.201000132 21290398

[B191] Stearns S. C. (1989). Trade-offs in life-history evolution. Funct. Ecol. 3, 259–268.

[B192] Steinert S. Levashina E. A. (2011). Intracellular immune responses of dipteran insects. Immunol. Rev. 240, 129–140. 10.1111/j.1600-065X.2010.00985.x 21349091

[B193] Storz P. (2005). Reactive oxygen species in tumor progression. Front. Biosci. 10, 1881–1896. 10.2741/1667 15769673

[B194] Stouthamer R. Breeuwer J. A. J. Hurst G. D. D. (1999). *Wolbachia pipientis*: microbial manipulator of arthropod reproduction. Annu. Rev. Microbiol. 53, 71–102. 10.1146/annurev.micro.53.1.71 10547686

[B195] Strand M. R. (2008). The insect cellular immune response. Insect Sci. 15, 1–14. 10.1111/j.1744-7917.2008.00183.x

[B196] Stuart J. A. Maddalena L. A. Merilovich M. Robb E. L. (2014). A midlife crisis for the mitochondrial free radical theory of aging. Longev. Healthspan 3, 4. 10.1186/2046-2395-3-4 24690218 PMC3977679

[B197] Suh Y.-A. Arnold R. S. Lassegue B. Shi J. Xu X. Sorescu D. (1999). Cell transformation by the superoxide-generating oxidase Mox1. Nature 401, 79–82. 10.1038/43459 10485709

[B198] Teixeira L. Ferreira A. Ashburner M. (2008). The bacterial symbiont *Wolbachia* induces resistance to RNA viral infections in *Drosophila melanogaster*. PLoS Biol. 6:e1000002. 10.1371/journal.pbio.1000002 19222304 PMC2605931

[B199] Tzou P. Ohresser S. Ferrandon D. Capovilla M. Reichhart J. M. Lemaitre B. (2000). Tissue-specific inducible expression of antimicrobial peptide genes in *Drosophila* surface epithelia. Immunity 13, 737–748. 10.1016/S1074-7613(00)00072-8 11114385

[B200] Valko M. Rhodes C. J. Moncol J. Izakovic M. Mazur M. (2006). Free radicals, metals and antioxidants in oxidative stress-induced cancer. Chem. Biol. Interact. 160, 1–40. 10.1016/j.cbi.2005.12.009 16430879

[B201] Virgin H. W. Levine B. (2009). Autophagy genes in immunity. Nat. Immunol. 10, 461–470. 10.1038/ni.1726 19381141 PMC2715365

[B202] Vollmer J. Schiefer A. Schneider T. Jülicher K. Johnston K. L. Taylor M. J. (2013). Requirement of lipid II biosynthesis for cell division in cell wall-less *Wolbachia*, endobacteria of arthropods and filarial nematodes. Int. J. Med. Microbiol. 303, 140–149. 10.1016/j.ijmm.2013.01.002 23517690

[B203] Voronin D. Cook D. A. N. Steven A. Taylor M. J. (2012). Autophagy regulates *Wolbachia* populations across diverse symbiotic associations. Proc. Natl Acad. Sci. U.S.A. 109, E1638–E1646. 10.1073/pnas.1203519109 22645363 PMC3382551

[B204] Voronin D. Guimarães A. F. Molyneux G. R. Johnston K. L. Ford L. Taylor M. J. (2014). *Wolbachia* lipoproteins: abundance, localisation and serology of *Wolbachia* peptidoglycan associated lipoprotein and the Type IV Secretion System component, VirB6 from *Brugia malayi* and *Aedes albopictus*. Parasit. Vectors 7, 462. 10.1186/s13071-014-0462-1 25287420 PMC4197220

[B205] Wang Y. Oberley L. W. Murhammer D. W. (2001). Evidence of oxidative stress following the viral infection of two lepidopteran insect cell lines. Free Radic. Biol. Med. 31, 1448–1455. 10.1016/S0891-5849(01)00728-6 11728817

[B206] Wang L. Zhou C. He Z. Wang Z.-G. Wang J.-L. Wang Y.-F. (2012). *Wolbachia* infection decreased the resistance of *Drosophila* to lead. PLoS ONE 7: e32643. 10.1371/journal.pone.0032643 22403688 PMC3293831

[B207] Weinert L. A. Araujo-Jnr E. V. Ahmed M. Z. Welch J. J. (2015). The incidence of bacterial endosymbionts in terrestrial arthropods. Proc. R. Soc. B 282, 20150249. 10.1098/rspb.2015.0249 25904667 PMC4424649

[B208] Werren J. H. Baldo L. Clark M. E. (2008). *Wolbachia*: master manipulators of invertebrate biology. Nat. Rev. Microbiol. 6, 741–751. 10.1038/nrmicro1969 18794912

[B209] Whitten M. M. A. Ratcliffe N. A. (1999). In vitro superoxide activity in the haemolymph of the West Indian leaf cockroach, *Blaberus discoidalis*. J. Insect. Physiol. 45, 667–675. 10.1016/S0022-1910(99)00039-6 12770352

[B210] Wong Z. S. Brownlie J. C. Johnson K. N. (2015). Oxidative stress correlates with *Wolbachia*-mediated antiviral protection in *Wolbachia*-*Drosophila* associations. Appl. Environ. Microbiol. 81, 3001–3005. 10.1128/AEM.03847-14 25710364 PMC4393424

[B211] Wong Z. S. Hedges L. M. Brownlie J. C. Johnson K. N. (2011). *Wolbachia* -mediated antibacterial protection and immune gene regulation in *Drosophila*. PLoS ONE 6: e25430. 10.1371/journal.pone.0025430 21980455 PMC3183045

[B212] Wu S.-C. Liao C.-W. Pan R.-L. Juang J.-L. (2012). Infection-induced intestinal oxidative stress triggers organ-to-organ immunological communication in *Drosophila*. Cell Host Microbe 11, 410–417. 10.1016/j.chom.2012.03.004 22520468

[B213] Xi Z. Gavotte L. Xie Y. Dobson S. L. (2008a). Genome-wide analysis of the interaction between the endosymbiotic bacterium *Wolbachia* and its *Drosophila* host. BMC Genomics 9:1. 10.1186/1471-2164-9-1 18171476 PMC2253531

[B214] Xi Z. Ramirez J. L. Dimopoulos G. (2008b). The *Aedes aegypti* Toll pathway controls dengue virus infection. PLoS Pathog. 4:e1000098. 10.1371/journal.ppat.1000098 18604274 PMC2435278

[B215] Yano T. Mita S. Ohmori H. Oshima Y. Fujimoto Y. Ueda R. (2008). Autophagic control of *Listeria* through intracellular innate immune recognition in *Drosophila*. Nat. Immunol. 9, 908–916. 10.1038/ni.1634 18604211 PMC2562576

[B216] Yi H.-Y. Chowdhury M. Huang Y.-D. Yu X.-Q. (2014). Insect antimicrobial peptides and their applications. Appl. Microbiol. Biotechnol. 98, 5807–5822. 10.1007/s00253-014-5792-6 24811407 PMC4083081

[B217] You H. Lee W. J. Lee W.-J. (2014). Homeostasis between gut-associated microorganisms and the immune system in *Drosophila*. Curr. Opin. Immunol. 30, 48–53. 10.1016/j.coi.2014.06.006 24997434

[B218] Yuk J.-M. Yoshimori T. Jo E.-K. (2012). Autophagy and bacterial infectious diseases. Exp. Mol. Med. 44, 99–108. 10.3858/emm.2012.44.2.032 22257885 PMC3296818

[B219] Yun J. Finkel T. (2014). Mitohormesis. Cell Metab. 19, 757–766. 10.1016/j.cmet.2014.01.011 24561260 PMC4016106

[B220] Zarse K. Schmeisser S. Groth M. Priebe S. Beuster G. Kuhlow D. (2012). Impaired insulin/IGF1 signaling extends life span by promoting mitochondrial L-proline catabolism to induce a transient ROS signal. Cell Metab. 15, 451–465. 10.1016/j.cmet.2012.02.013 22482728 PMC4844853

[B221] Zélé F. Nicot A. Duron O. Rivero A. (2012). Infection with *Wolbachia* protects mosquitoes against *Plasmodium*-induced mortality in a natural system. J. Evol. Biol. 25, 1243–1252. 10.1111/j.1420-9101.2012.02519.x 22533729

[B222] Zélé F. Nicot A. Berthomieu A. Weill M. Duron O. Rivero A. (2014). *Wolbachia* increases susceptibility to *Plasmodium* infection in a natural system. Proc. R. Soc. B. 281, 20132837. 10.1098/rspb.2013.2837 24500167 PMC3924077

[B223] Zhang Y.-K. Ding X.-L Rong X. Hong X.-Y. (2015). How do hosts react to endosymbionts? A new insight into the molecular mechanisms underlying the *Wolbachia*-host association. Insect Mol. Biol. 24, 1–12. 10.1111/imb.12128 25224730

[B224] Zouache K. Voronin D. Tran-Van V. Mousson L. Failloux A.-B. Mavingui P. (2009). Persistent *Wolbachia* and cultivable bacteria infection in the reproductive and somatic tissues of the mosquito vector *Aedes albopictus*. PLoS ONE 4:e6388. 10.1371/journal.pone.0006388 19633721 PMC2712238

[B225] Zug R. Hammerstein P. (2012). Still a host of hosts for *Wolbachia*: analysis of recent data suggests that 40% of terrestrial arthropod species are infected. PLoS ONE 7:e38544. 10.1371/journal.pone.0038544 22685581 PMC3369835

[B226] Zug R. Hammerstein P. (2015). Bad guys turned nice? A critical assessment of *Wolbachia* mutualisms in arthropod hosts. Biol. Rev. 90, 89–111. 10.1111/brv.12098 24618033

[B227] Zug R. Koehncke A. Hammerstein P. (2012). Epidemiology in evolutionary time: the case of *Wolbachia* horizontal transmission between arthropod host species. J. Evol. Biol. 25, 2149–2160. 10.1111/j.1420-9101.2012.02601.x 22947080

[B228] Zuk M. Stoehr A. M. (2002). Immune defense and host life history. Am. Nat. 160, S9–S22. 10.1086/342131 18707455

